# Oncogenic *PIK3CA* mutations reprogram glutamine metabolism in colorectal cancer

**DOI:** 10.1038/ncomms11971

**Published:** 2016-06-20

**Authors:** Yujun Hao, Yardena Samuels, Qingling Li, Dawid Krokowski, Bo-Jhih Guan, Chao Wang, Zhicheng Jin, Bohan Dong, Bo Cao, Xiujing Feng, Min Xiang, Claire Xu, Stephen Fink, Neal J. Meropol, Yan Xu, Ronald A. Conlon, Sanford Markowitz, Kenneth W. Kinzler, Victor E. Velculescu, Henri Brunengraber, Joseph E. Willis, Thomas LaFramboise, Maria Hatzoglou, Guo-Fang Zhang, Bert Vogelstein, Zhenghe Wang

**Affiliations:** 1 Department of Genetics and Genome Sciences, Case Western Reserve University, 10900 Euclid Avenue, Cleveland, Ohio 44106, USA; 2 Case Comprehensive Cancer Center, Case Western Reserve University, 10900 Euclid Avenue, Cleveland, Ohio 44106, USA; 3 Ludwig Center and Howard Hughes Medical Institute, Sidney Kimmel Comprehensive Cancer Center at Johns Hopkins, Baltimore, Maryland 21231, USA; 4 Department of Molecular Cell Biology, The Weizmann Institute of Science, Rehovot 76100, Israel; 5 Department of Nutrition, Case Western Reserve University, 10900 Euclid Avenue, Cleveland, Ohio 44106, USA; 6 Department of Pharmacology, School of Medicine, Case Western Reserve University, 10900 Euclid Avenue, Cleveland, Ohio 44106, USA; 7 Department of Biochemistry and Molecular Biology, Wannan Medical College, Wuhu 241000, China; 8 Department of Pharmacognosy, School of Pharmacy, Third Military Medical University, Chongqing 400038, China; 9 Department of Pharmacy, Suzhou Health College, Suzhou, Jiangsu 215009, China; 10 Hathaway Brown School, 19600 North Park Boulevard, Shaker Heights, Ohio 44122, USA; 11 Department of Medicine, University Hospitals Case Medical Center, Case Western Reserve University, 10900 Euclid Avenue, Cleveland, Ohio 44106, USA; 12 Department of Chemistry, Cleveland State University, 2121 Euclid Avenue, Cleveland, Ohio 44115, USA; 13 Department of Pathology, University Hospitals Case Medical Center, Case Western Reserve University, 10900 Euclid Avenue, Cleveland, Ohio 44106, USA

## Abstract

Cancer cells often require glutamine for growth, thereby distinguishing them from most normal cells. Here we show that *PIK3CA* mutations reprogram glutamine metabolism by upregulating glutamate pyruvate transaminase 2 (GPT2) in colorectal cancer (CRC) cells, making them more dependent on glutamine. Compared with isogenic wild-type (WT) cells, *PIK3CA* mutant CRCs convert substantially more glutamine to α-ketoglutarate to replenish the tricarboxylic acid cycle and generate ATP. Mutant p110α upregulates *GPT2* gene expression through an AKT-independent, PDK1–RSK2–ATF4 signalling axis. Moreover, aminooxyacetate, which inhibits the enzymatic activity of aminotransferases including GPT2, suppresses xenograft tumour growth of CRCs with *PIK3CA* mutations, but not with WT *PIK3CA*. Together, these data establish oncogenic *PIK3CA* mutations as a cause of glutamine dependency in CRCs and suggest that targeting glutamine metabolism may be an effective approach to treat CRC patients harbouring *PIK3CA* mutations.

## Abstract (web summary)

Cancer cells rely on glutamine to replenish the TCA cycle. Here, the authors show that oncogenic *PIK3CA* mutations drive this metabolic rewiring in colorectal cancer cells by up-regulating glutamate pyruvate transaminase expression, thus increasing sensitivity to glutamine starvation.

## Graphical abstract

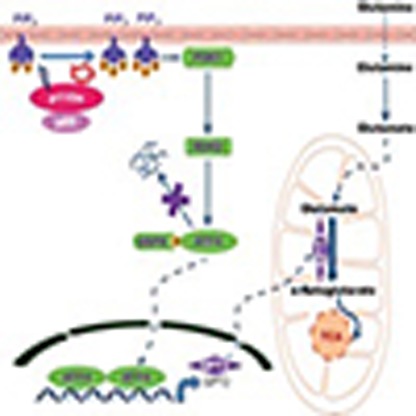

Cancer cells are distinguished from most normal cells by metabolic reprogramming, including phenomena termed the Warburg effect and glutamine dependency^1^,^2^. Normally, glucose is converted to acetyl-CoA, which enters the tricarboxylic acid (TCA) cycle. Cancer cells, however, convert glucose to lactate even in the presence of oxygen (Warburg effect) and utilize glutamine to replenish the TCA cycle^3^. To enter the TCA cycle, glutamine is first deaminated by glutaminases (GLSs) to glutamate^4^. Glutamate is then converted to α-ketoglutarate (α-KG), which is a substrate in the TCA cycle. Three groups of enzymes can convert glutamate to α-KG: (1) glutamate pyruvate transaminases (GPTs); (2) glutamate oxaloacetate transaminases (GOTs); and (3) glutamate dehydrogenases (GLUDs)^4^. The metabolic products of glutamine are utilized both to produce ATP and to synthesize macromolecules in the promotion of tumour growth^4^. Although glutamine is a non-essential amino acid, it has long been recognized that glutamine is a required supplement for culturing cancer cells.

Many oncogenes and tumour suppressors impact glutamine metabolism^4^. Myc overexpression affects cellular glutamine levels by inducing the transcription of GLS1 and the glutamine transporter SLC1A5 (a.k.a. ASCT2)^5^,^6^. In contrast, SLC1A5 expression is repressed by the Rb tumour suppressor^7^, whereas GLS2 was identified as a transcriptional target of p53 (ref. 8). In addition, it has been shown that p53 represses the expression of malic enzymes ME1 and ME2, thereby regulating glutamine-dependent NADPH production^9^. A recent study showed that loss of tumour suppressor von hippel-lindau tumor suppressor (VHL) renders renal cell carcinomas sensitive to glutamine deprivation through hypoxia induced factor (HIF)-induced metabolic reprogramming^10^. Moreover, K-ras upregulates the aminotransferase GOT1 (ref. 11). Though all of these mechanisms impact the production or degradation of glutamine or its metabolites, the mechanisms by which many cancer cells become dependent on glutamine are still unknown or actively debated.

*PIK3CA* encodes the catalytic subunit of phosphatidylinositol 3-kinase α (PI3Kα), which plays a key role in regulating cell proliferation, survival and motility^12^. PIK3α consists of a catalytic subunit p110α, and one of several regulatory subunits (a major one being p85α)^13^. On growth factor stimulation, p85 is recruited to phosphorylated receptor protein kinases and adaptor proteins, thereby activating PI3Kα. Activated PI3Kα converts phosphatidylinositol-4,5-biophosphate (PIP2) to phosphatidylinositol-3,4,5-triphosphate (PIP3). The second message PIP3 then activates PDK1 and AKT signalling. *PIK3CA* is mutated in a wide variety of human cancers including ∼30% of colorectal cancers (CRCs)^14^. Recent large-scale sequencing of human cancer genomes reveals that *PIK3CA* is the most frequently mutated oncogene in human cancer^15^. However, the fact that *PIK3CA* mutations can reprogram cancer metabolism, as demonstrated herein, was previously unknown.

We report that *PIK3CA* mutations render CRCs more sensitive to glutamine deprivation by upregulation of GPT2, an enzyme involved in glutamine metabolism. We further demonstrate that mutant p110α increases GPT2 gene expression through an AKT-independent signalling pathway. Moreover, we show that aminooxyacetate (AOA), a compound that inhibits enzymatic activity of aminotransferases, suppresses xenograft tumour growth of CRCs with *PIK3CA* mutations, but not with wild-type (WT) *PIK3CA*. These results suggest that reprogramming glutamine metabolism is crucial for the oncogenic function of *PIK3CA* mutations and that targeting glutamine metabolism may be an effective approach to treating CRC patients harbouring tumour mutations of this gene.

## Results

### *PIK3CA* mutations render CRC cells dependent on glutamine

**Figure 1:**
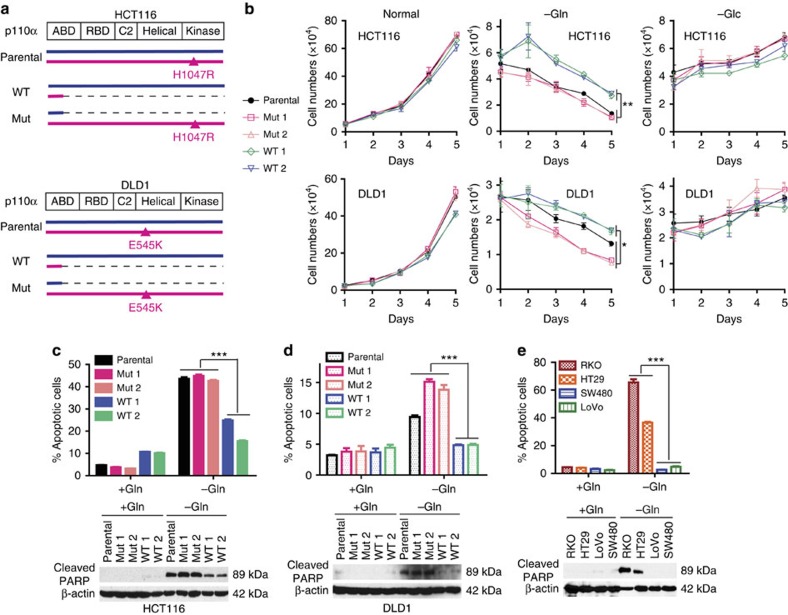
*PIK3CA* mutation renders CRC cells more sensitive to glutamine deprivation. (**a**) *PIK3CA* parental, WT and mutant allele configurations of CRC lines. (**b**) *PIK3CA* mutation makes cells sensitive to glutamine, but not to glucose, deprivation. Numbers of cells of the indicated genotypes were determined in medium with both glucose and glutamine (normal), medium without glutamine (−Gln) and medium without glucose (−Glc) at indicated time points. Parental cells (HCT116 and DLD1) and two independently derived mutant (Mut 1 and Mut 2) or WT (WT 1 and WT 2) clones for each parental line were analysed. (**c**,**d**) Glutamine deprivation induces more apoptosis in cells mutant for *PIK3CA*. HCT116 (**c**) and DLD1 (**d**) cells of the indicated genotypes were grown with or without 2 mM glutamine for 72 h. Cell apoptosis was measured as per cent sub-G1 cells and with cleaved PARP protein. (**e**) Glutamine deprivation induces more apoptosis in *PIK3CA* mutant CRC cell lines. CRC cell lines WT or mutant for *PIK3CA* were grown with or without glutamine for 72 h. Cell apoptosis was measured by profiling sub-G1 cells and cleaved PARP. *PIK3CA* mutant cell lines: RKO and HT29; WT *PIK3CA* cell lines: LoVo and SW480. Data are presented as mean±s.e.m. of three independent cultures. *P<0.05; **P<0.01; ***P<0.001, *t*-test.

Most *PIK3CA* mutations are clustered in two hotspots, with H1047R in the kinase domain and E545K in the helical domain the most common mutations^16^. We set out to determine whether *PIK3CA* mutations reprogram cell metabolism in CRCs. The CRC cell line HCT116 harbours a heterozygous H1047R mutation, whereas DLD1 CRC cells have a heterozygous E545K mutation (Fig. 1a). We exploited isogenic derivatives of these cell lines in which either the WT or mutant allele of *PIK3CA* is knocked out (Fig. 1a)^17^. The clones in which the mutant allele had been disrupted (and the WT allele was intact) were called ‘WT' (Fig. 1a), whereas the clones in which only the WT allele had been disrupted (and the mutant allele was intact) were called ‘mutant' (Mut, Fig. 1a)^17^. As reported previously^17^, the parental cells and their derived knockout clones grew at similar rate under normal conditions in the presence of both glucose and glutamine (Fig. 1b). However, in medium without glutamine, both parental cells and the two independently derived *PIK3CA* mutant clones died faster than did two independently derived *PIK3CA* WT clones (Fig. 1b). This relative sensitivity to glutamine deprivation dependent on *PIK3CA* status was observed in both HCT116 and DLD1 (Fig. 1b). Consistently, glutamine deprivation induced more apoptotic cells in the mutant clones than in the WT clones as assayed by percentages of sub-G1 cells and amounts of cleaved PARP (Fig. 1c,d). In contrast, differential sensitivity to deprivation of glucose was not found (Fig. 1b and Supplementary Fig. 1a). Surprisingly, these cell lines still grew equally well, although slowly, in the absence of glucose (Fig. 1b). Because recent studies showed that lung cancer cells can become independent of glucose by upregulation of mitochondrial phosphoenolpyruvate carboxykinase (PCK2), which generates phosphoenolpyruvate from glutamine to generate ribose for nucleic acid synthesis^18^,^19^, we set out to determine whether PCK2 was upregulated in the HCT116 and DLD1 cell lines when deprived of glucose. As shown in Supplementary Fig. 1b, PCK2 was indeed upregulated in both cell lines.

To determine if metabolic reprogramming by *PIK3CA* mutation was generalizable, we tested glutamine sensitivity in two CRC cell lines with *PIK3CA* mutations (RKO (containing a *PIK3CA* H1047R mutation) and HT29 (containing a *PIK3CA* P449T mutation)) and two CRC cell lines with WT *PIK3CA* (SW480 and LOVO). As shown in Fig. 1e, glutamine deprivation induced significantly more apoptotic cells in the *PIK3CA* mutant cell lines than in the WT *PIK3CA* cell lines.

Since KRAS increases glutamine metabolism in pancreatic cancers^11^, we set out to determine if *KRAS* mutations affects glutamine dependency in CRC cells. HCT116 and DLD1 cells have heterozygous oncogenic *KRAS* mutations. Using isogenic HCT116 and DLD1 clones, with either WT or mutant KRAS knockout^20^, we showed that the cells did not have differential sensitivity to glutamine dependent on *KRAS* genotype (Supplementary Fig. 1c,d). Taken together, the data suggest that *PIK3CA* mutations may be responsible for CRC cell dependence on glutamine for optimal growth.

### *PIK3CA* mutations upregulates GPT2 expression

**Figure 2:**
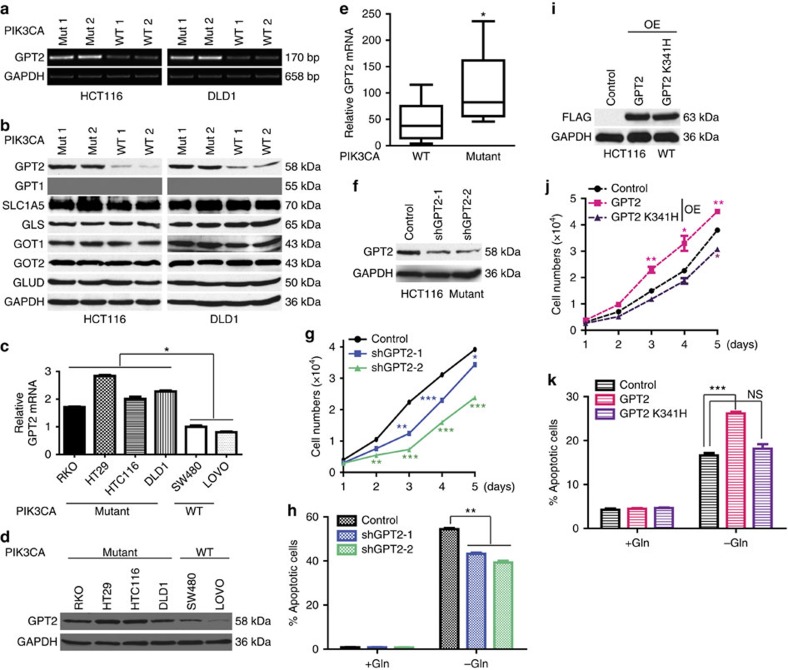
*PIK3CA* mutation upregulates GPT2 which renders CRC dependent on glutamine. (**a**) GPT2 mRNA levels are higher in *PIK3CA* mutant clones. RT–PCR analyses of the indicated genes in the HCT116 and DLD1 *PIK3CA* mutant and WT clones. (**b**) GPT2 protein levels are higher in cells with *PIK3CA* mutations. Cell lysates of WT and mutant clones were blotted with the indicated antibodies. SLC1A5: glutamine transporter. (**c**,**d**) GPT2 expression is upregulated in *PIK3CA* mutant CRC cell lines. qRT–PCR (**c**) and western blot of GPT2 protein in the indicated cell lines (**d**). (**e**) GPT2 mRNA levels are higher in PIK3CA mutant tumours. qRT–PCR analyses of GPT2 in tumours with no mutations in *PIK3CA* or in *PTEN*, *PDK1*, *AKT*s and *IRS* (*n*=10) versus tumours with *PIK3CA* mutations (*n*=10). Data are plotted as whiskers (min to max). (**f**–**h**) GPT2 is needed for growth and for glutamine dependency in *PIK3CA* mutant cells. GPT2 was knocked down with two independent shRNAs in a HCT116 *PIK3CA* mutant clone (Mut 1). Stable pools were selected for further analysis. Western blot of GPT2 in control and GPT2 knockdown clones (**f**). Two thousand cells were seeded in 96-well plates and grown under normal culture conditions, and cell numbers were counted 5 consecutive days (**g**). Control and GPT2 knockdown clones were grown with or without glutamine for 72 h. Cell apoptosis was quantified (**h**). (**i**–**k**) GPT2 is sufficient for growth and for creating glutamine sensitivity in *PIK3CA* WT cells. A HCT116 *PIK3CA* WT clone (WT 1) was transfected with empty vector (control), or a FLAG-tagged WT GPT2 (GPT2) or a FLAG-tagged inactive GPT2 mutant (GPT2 K341H). Stable pools were selected for further analysis. Western blot analyses of transfected FLAG-GPT2 protein levels (**i**); cell proliferation under normal culture conditions (**j**); control and GPT2 overexpression (OE) cell pools were grown with or without glutamine for 72 h. Cell apoptosis was quantified (**k**). Data are presented as mean±s.e.m. of three independent cultures. *P<0.05; **P<0.01; ***P<0.001; NS, not significant; *t*-test.

To determine whether *PIK3CA* mutations regulate enzymes involved in glutamine metabolism, we performed serial analysis of gene expression (SAGE)^21^ on the isogenic cell lines described above (Supplementary Data 1). Interestingly, the expression levels of mitochondrial GPT2, which converts glutamate to α-KG, were upregulated in both HCT116 and DLD1 *PIK3CA* mutant clones compared with the WT clones. This observation was confirmed by both reverse transcription–PCR (RT–PCR) and western blot analyses of the clones (Fig. 2a,b and Supplementary Fig. 2a). None of the other enzymes primarily involved in glutamine metabolism, including GLS, GPT1, GOT1, GOT2, GLUD or the glutamine transporter SLC1A5, exhibited differential protein levels among the *PIK3CA* mutant and WT clones (Fig. 2b). Moreover, both GPT2 RNA and protein levels were higher in the four different *PIK3CA* mutant lines (HCT116, DLD1, RKO and HT29) than in the two *PIK3CA* WT cell lines (SW480 and LOVO) (Fig. 2c,d and Supplementary Fig. 2b). In agreement with the notion that *KRAS* mutations do not play a role in glutamine dependency in CRC, none of the enzymes showed differential expression levels in the *KRAS* WT and mutant clones (Supplementary Fig. 2c).

We next tested if GPT2 expression is upregulated in CRC tumour specimens with *PIK3CA* mutations. We measured GPT2 RNA levels by quantitative RT–PCR (qRT–PCR) in 20 human CRC patient tumours, which we had previously sequenced the exomes (10 tumours with *PIK3CA* mutations and 10 tumours without mutations in the *PIK3CA* pathway)^22^. As shown in Fig. 2e, expression levels of GPT2 were significantly higher in the tumours with PIK3CA mutation than in the tumours with WT *PIK3CA*. To further extend this observation, we mined the TCGA data set of CRCs with RNA-seq data. As shown in Supplementary Fig. 2d, CRCs with *PIK3CA* mutation express significantly higher levels of GPT2 than CRCs with WT *PIK3CA*. Moreover, for those tumours with matched normal tissues, GPT2 expression is upregulated in nearly all of them (Supplementary Fig. 2e).

To determine whether the upregulation of GPT2 makes *PIK3CA* mutant cells dependent on glutamine, we knocked down GPT2 in the HCT116 *PIK3CA* mutant clone using two independent shRNAs (Fig. 2f). Because independently derived *PIK3CA* mutant and WT clones exhibited near-identical phenotypes, we chose one *PIK3CA* mutant clone (Mut 1) and one *PIK3CA* WT clone (WT 1) for in-depth analyses. Compared with cells with a control shRNA, the GPT2 knockdown cells grew more slowly under normal growth conditions (Fig. 2g), but they were less sensitive to glutamine deprivation as assayed by cell apoptosis (Fig. 2h). These results were further validated with GPT2 CRISPR/Cas9 knockouts in HCT116 cells (Supplementary Fig. 2f–i). Although GPT2 knockout cells grew slower than the parental cells, they were less sensitive to glutamine deprivation (Supplementary Fig. 2h,i). Moreover, reconstitution of the knockout cells by restoration of GPT2 expression made these cells grow faster and more sensitive to glutamine deprivation (Supplementary Fig. 2j–l). In contrast, knockdown of GPT2 in the HCT116 *PIK3CA* WT clone (WT 1) had no effect on their sensitivity to glutamine deprivation or proliferation under normal culture conditions (Supplementary Fig. 2m–o). Importantly, overexpression of GPT2 in the WT clone made it grow faster under normal culture conditions, and more sensitive to glutamine deprivation (Fig. 2i–k and Supplementary Fig. 2p). However, overexpression of an enzymatic inactive mutant GPT2 in the WT clone had no impact on either cell proliferation or sensitivity to glutamine deprivation (Fig. 2i–k). In aggregate, these data demonstrate that *PIK3CA* mutations render CRC cells more sensitive to glutamine withdrawal through upregulation of GPT2.

### AOA inhibits the growth of *PIK3CA*-mutant CRCs *in vivo*

**Figure 3:**
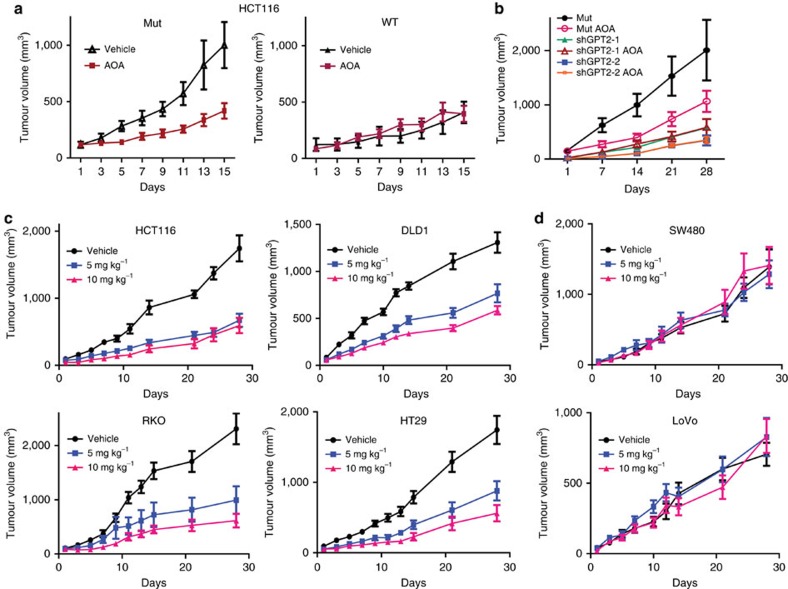
AOA inhibits xenograft tumour growth of *PIK3CA* mutant CRCs but not *PIK3CA* WT CRCs. (**a**) AOA inhibits growth of xenograft formed by HCT116 *PIK3CA* mutant clones but not *PIK3CA* WT clones. Three million cells were injected subcutaneously and bilaterally into athymic nude mice. Once tumours reached an average size of 100 mm^3^, 10 mg kg^−1^ of AOA was injected by intraperitoneal every day for 2 weeks. (**b**) AOA does not inhibit growth of xenograft tumours formed by GPT2 knockdown cells generated from a HCT116 *PIK3CA* mutant clone (Mut 1). Xenograft tumours of indicated cells were established and treated as described in **a** for 4 weeks. (**c**) AOA inhibits growth of xenograft tumours formed by the indicated four CRC cell lines harbouring *PIK3CA* mutations. (**d**) AOA does not inhibit growth of xenograft tumours formed by the indicated two CRC cell lines with WT *PIK3CA*. *N*=5 mice in each experimental group. Data are presented as mean±s.e.m. For HCT116 *PIK3CA* mutant clones, HCT116, DLD1, RKO and HT29, AOA treatment significantly inhibits xenograft tumour growth. *P*<0.001, two-way analysis of variance analysis.

AOA inhibits the enzymatic activity of aminotransferases, including GPT2 (ref. 23). As shown in Supplementary Fig. 3a, both HCT116 and DLD1 *PIK3CA* mutant clones were more sensitive to AOA treatment than the WT clones in tissue culture. Moreover, AOA significantly inhibited the growth of HCT116 and DLD1 *PIK3CA* mutant clones when xenografted into nude mice (Fig. 3a and Supplementary Fig. 3b). In contrast, AOA had no effect on isogenic *PIK3CA* WT xenograft tumours (Fig. 3a and Supplementary Fig. 3b), although those tumours grew more slowly than their mutant counterparts in the absence of AOA (Fig. 3a and Supplementary Fig. 3b). Importantly, we further demonstrated that AOA inhibited growth of *PIK3CA* mutant xenograft tumours by targeting GPT2, because tumours established from the two GPT2 knockdown cell pools were insensitive to AOA treatment, although they grew much slower than HCT116 *PIK3CA* mutant tumours expressing a control shRNA (Fig. 3b). AOA treatment consistently reduced amounts of α-KG and alanine, but increased amounts of glutamate and pyruvate, in the HCT116 *PIK3CA* mutant tumours (Supplementary Fig. 3c).

To test whether our observations with the genetically engineered cell lines were generalizable, we expanded our xenograft study to a panel of CRC cell lines. AOA inhibited xenograft tumour growth of four *PIK3CA* mutant CRC cell lines (HCT116 (parental cells), DLD1 (parental cells), RKO and HT29; Fig. 3c). In contrast, AOA had no effect on xenograft tumour growth of two WT *PIK3CA* CRC cell lines (SW480 and LOVO; Fig. 3d). No weight loss was observed for the mice that were treated with AOA (Supplementary Fig. 3d), suggesting that the doses of AOA used in the experiments had minimal toxicity.

### ATF4 regulates transcription of GPT2

**Figure 4:**
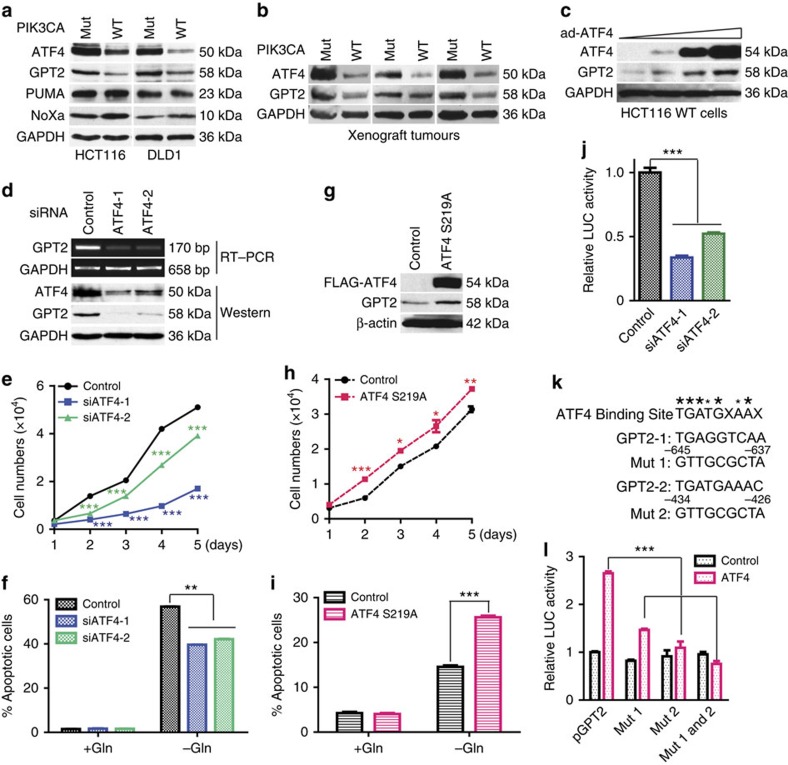
ATF4 activates GPT2 transcription in *PIK3CA* mutant cells. (**a**,**b**) ATF4 protein levels correlate with that of GPT2 in *PIK3CA* WT and mutant clones. Western blot of lysates of cultured cells (**a**) or lysates of the xenograft tumours formed by the HCT116 clones (**b**). (**c**) Overexpression of ATF4 in HCT116 *PIK3CA* WT increases GPT2 protein levels. HCT116 *PIK3CA* WT cells were infected an adenovirus expressing ATF4 (ad-ATF4). (**d**) Knockdown of ATF4 in the HCT116 *PIK3CA* mutant clone decreases GPT2 mRNA and protein levels. (**e**) Knockdown of ATF4 reduces proliferation of HCT116 *PIK3CA* mutant cells. Scrambled (control) siRNA and two independent siRNAs were transfected into HCT116 *PIK3CA* mutant clone. (**f**) Knockdown of ATF4 in the HCT116 *PIK3CA* mutant clone renders the cells less sensitive to glutamine deprivation. (**g**) Overexpression of ATF4 S219A stabilization mutant in the HCT116 *PIK3CA* WT clone increases GPT2 protein levels. A FLAG-tagged ATF4 S219A construct was transfected in HCT116 *PIK3CA* WT cells and stable pools were selected. Cell lysates were blotted with the indicated antibodies. (**h**) Overexpression of stabilized ATF4 S219A mutant in the HCT116 *PIK3CA* WT cells increases cell proliferation. (**i**) Overexpression of stabilized ATF4 S219A mutant in the HCT116 *PIK3CA* WT clone renders it more sensitive to glutamine deprivation. Control and ATF4 S219A overexpression cells were grown with or without glutamine for 72 h. Apoptotic cells were quantified. (**j**) Knockdown of ATF4 in a HCT116 *PIK3CA* mutant clone decreases activity of a GPT2 promoter transcriptional reporter. GPT2 luciferase reporter plasmid was co-transfected with the indicated siRNA and a plasmid expressing β-galactosidase as internal control. Luciferase activities were assayed 48 h post transfection. (**k**) Sequence of two putative ATF4-binding sites in the GPT2 promoter, which were predicted by TFseach v1.3 and mutant sequences that abolish ATF4 binding. (**l**) Mutation of ATF4-binding sites reduces GPT2 promoter reporter expression. Empty vector or vector expressing ATF4 were co-transfected with the indicated reporter plasmids in HCT116 *PIK3CA* WT cells. Data are presented as mean±s.e.m. of three independent experiments. *P<0.05; **P<0.01; ***P<0.001.

Given that p110α is not a transcription factor, a key question raised by these data is how mutant p110α transduces the signals that activate GPT2 transcription. To address this question, we evaluated ATF4, as recent studies reported that ATF4 is involved in glutamine metabolism^6^,^24^. Interestingly, ATF4 protein levels mirrored GPT2 protein levels in the *PIK3CA* mutant and WT clones (Fig. 4a). This correlation was maintained in xenograft tumours (Fig. 4b). Because ATF4 induces expression of pro-apoptotic BH3-only proteins PUMA and Noxa in neuroblastoma cells^6^, we also measured levels of these two proteins in the *PIK3CA* WT and mutant clones. However, PUMA and Noxa protein levels were not correlated with ATF4 levels (Fig. 4a), suggesting that the regulation of the two pro-apoptotic proteins by ATF4 may be cell-type specific or controlled by other factors.

As shown in Fig. 4c, overexpression of ATF4 in the HTC116 *PIK3CA* WT clone increased GPT2 protein levels in a dose-dependent manner. Conversely, knockdown of ATF4 in the HCT116 *PIK3CA* mutant clone by two different siRNAs reduced both mRNA and protein levels of GPT2 (Fig. 4d). In contrast, knockdown of ATF4 did not affect the expression of other enzymes involved in glutamine metabolism, including SLC1A5, GLS1, GOT1, GOT2 and GLUD1 (Supplementary Fig. 4a). Similar results were observed in both HCT116 and DLD1 *PIK3CA* mutant clones (Fig. 4d and Supplementary Fig. 4a,b). Importantly, as in the case with GPT2, knockdown of ATF4 rendered *PIK3CA* mutant cells less proliferative under normal culture conditions, but more resistant to glutamine deprivation (Fig. 4e,f). Conversely, overexpression of a proteasome-resistant ATF4 S219A mutant in HCT116 WT cells increased GPT2 protein levels and cell proliferation under normal culture conditions (Fig. 4g,h), but made them more sensitivity to glutamine deprivation (Fig. 4i). These results suggest that the mutant p110α–ATF4–GPT2 axis regulates glutamine metabolism.

To determine whether ATF4 activates GPT2 gene transcription directly, we cloned 1.5 kb of genomic DNA upstream of the transcription start site of the GPT2 gene into a luciferase reporter plasmid. As shown in Fig. 4j, knockdown of ATF4 in the HCT116 *PIK3CA* mutant clone reduced transcriptional activity of the GPT2 reporter. Examining the DNA sequence in the 1.5-kb genomic DNA fragment of GPT2, we found two motifs matching ATF4 consensus-binding sites (Fig. 4k). Mutating either site individually or both together significantly diminished ATF4-mediated transcriptional activity (Fig. 4l).

### Mutant p110α stabilizes the ATF4 protein

**Figure 5:**
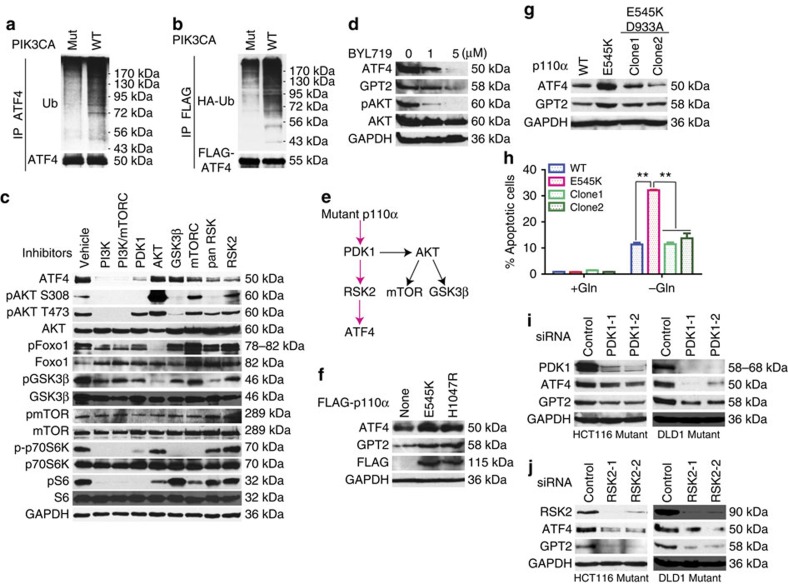
The p110α–PDK1–RSK2 signalling axis regulates ATF4 protein stability. (**a**,**b**) ATF4 ubiquitination levels are higher in a HCT116 WT clone than the *PIK3CA* mutant clone. HCT116 *PIK3CA* WT and mutant cells were treated with 5 μM MG132 for 6 h. Endogenous ATF4 proteins were immunoprecipitated and blotted with an anti-ubiquitin antibody (**a**). FLAG-tagged ATF4 and human influenza hemagglutinin (HA)-tagged ubiquitin plasmids were co-transfected into HCT116 *PIK3CA* WT and mutant cells. FLAG-tagged ATF4 was immunoprecipitated and blotted with anti-HA antibody to detect HA-tagged ubiquitin (**b**). (**c**,**d**) Inhibitors of PI3K, PDK1 and RSK2 reduce ATF4 protein levels in a HCT116 *PIK3CA* mutant clone. Inhibitors include 10 μM LY294002 (pan-PI3K inhibitor), 1 or 5 μM BYL-719 (p110α-specific inhibitor), 5 μM BEZ235 (PI3K/mTOR dual-specificity inhibitor), 6 μM GSK2334470 (PDK1 inhibitor), 10 μM GSK690693 (AKT inhibitor), 10 μM CHIR-99021 (GSK3β inhibitor), 10 μM rapamycin (mTOR inhibitor), 10 μM BI-D1870 (pan-RSK inhibitor) and 10 μM FMK (RSK2 inhibitor). Cells were treated with vehicle or each inhibitor for 12 h and lysates were blotted with the indicated antibodies. (**e**) A schematic of the p110α signalling pathway that regulates ATF4 protein stability. (**f**) Overexpression of oncogenic p110α mutants increases ATF4 and GPT2 protein levels. The indicated constructs were transfected into HCT116 WT cells. Cell lysates were blotted with the indicated antibodies. (**g**) A kinase-dead mutation combined with the p110α E545K mutation in the same allele reduces protein levels of ATF4 and GPT2. A *PIK3CA* D993A kinase-dead mutant was knocked into a DLD1 *PIK3CA* mutant clone as described in Supplementary Fig. 5e,f. Cell lysates were blotted with the indicated antibodies. (**h**) Genetic inactivation of p110α enzymatic activity renders DLD1 *PIK3CA* oncogenic mutant cells less sensitive to glutamine deprivation. Clones of the indicated genotypes were grown with or without glutamine for 72 h. Apoptotic cells were quantified. Data presented as mean±s.e.m. of three independent cultures. **P<0.01, *t*-test. (**i**) Knockdown of PDK1 by two independent siRNAs reduces ATF4 and GPT2 protein levels in both HCT116 and DLD1 *PIK3CA* mutant clones. (**j**) Knockdown of RSK2 by two independent siRNAs reduces ATF4 and GPT2 protein levels.

We next sought to determine how ATF4 is differentially regulated in *PIK3CA* mutant and WT cells. We first showed that ATF4 mRNA levels were similar in isogenic *PIK3CA* mutant and WT cell clones (Supplementary Fig. 4c). Translation of ATF4 mRNA is known to be induced by stress through phospho-eIF2α (p-eIF2α)-dependent translation initiation of upstream open reading frames (uORF)^25^. To investigate whether mutant p110α upregulates ATF4 protein levels through this mechanism, we examined p-eIF2α levels in the *PIK3CA* mutant and WT clones. As shown in Supplementary Fig. 4d, similar levels of p-eIF2α were observed in the *PIK3CA* mutant and WT clones under normal culture conditions. This result was in agreement with the uORF reporter assays indicating that the uORF initiation activity of ATF4 was similar in the HCT116 *PIK3CA* mutant and WT cells (Supplementary Fig. 4e). Moreover, polysome profiles of ATF4 mRNA were similar in HCT116 *PIK3CA* mutant and WT clones (Supplementary Fig. 4f). We also tested whether mutant p110α affected ATF4 protein stability by regulating its ubiquitination. As shown in Fig. 5a,b, the ubiquitination levels of both endogenous and overexpressed ATF4 were much higher in the HCT116 *PIK3CA* WT clone than in the mutant cells. Consistent with this result, treatment of HCT116 and DLD1 *PIK3CA* WT cells with a proteasome inhibitor MG132 resulted in increased protein levels of ATF4 and GPT2 (Supplementary Fig. 4g). MG132 treatment also increased apoptosis in these cells in the presence or absence of glutamine (Supplementary Fig. 4h)—presumably because of effects on other pathways—preventing further investigation of MG132 treatments.

As expected, exposure to a pan PI3K inhibitor (LY294002), a p110α-specific inhibitor (BYL-719), a PI3K/mTOR dual inhibitor (BEZ235) and a PDK1 inhibitor (GSK2334470), each reduced ATF4 protein levels (Fig. 5c,d). Surprisingly, neither an AKT inhibitor (GSK690693) nor an inhibitor (CHIR-99021) of GSK3β (a known downstream effector of AKT) had any effect on ATF4 protein levels (Fig. 5c). In accord with this, expression of a constitutively active form of AKT1 (myristoylated AKT1) in the *PIK3CA* WT clone did not affect ATF4 or GPT2 proteins levels (Supplementary Fig. 5a). As a control for these experiments, we found that myristoylated AKT1 did increase the phosphorylation level of AKT and FOXO1 (Supplementary Fig. 5a), a well-known AKT kinase target. Moreover, overexpression of myristoylated AKT1 did not make HCT116 *PIK3CA* WT cells more sensitive to glutamine deprivation (Supplementary Fig. 5b,c). Although mTOR was activated in *PIK3CA* mutant cells when deprived of glutamine (Supplementary Fig. 5d), our data suggest that the activation of mTOR does not determine glutamine sensitivity in these cells, because the overexpression of myristoylated AKT1 increased mTOR activity in the *PIK3CA* WT in the absence of glutamine (Supplementary Fig. 5a).

Interestingly, ATF4 is reported to be a substrate of RSK2 (ref. 26). Although it is not as well-known an effector of PI3K as AKT, PDK1 also regulates the RSK2 kinase^27^. Indeed, both a pan RSK inhibitor (BI-D1870) and a more selective RSK2 inhibitor (FMK) reduced ATF4 protein levels in the HCT116 *PIK3CA* mutant clone (Fig. 5c). In contrast, the inhibitors of p110α, PDK1 and RSK2 had little or no effect on ATF4 protein level in the HCT116 *PIK3CA* WT clone (Supplementary Fig. 4i), suggesting that the activity of the p110α–PDK1–RSK2–ATF4 pathway is low in these cells. Together, our data suggest a mutant p110α-activated PDK1–RSK2 pathway that regulates ATF4 protein levels (Fig. 5e, indicated by the red arrows).

To confirm the results obtained with the inhibitors, we first overexpressed p110α E545K and H1047R mutant constructs in the HCT116 *PIK3CA* WT clone. We found that overexpression of mutant p110α increased both ATF4 and GPT2 protein levels (Fig. 5f). We then attempted to assess the generality of these observations and ascertain whether the lipid kinase activity of p110α is required for the mutant p110α signalling pathway to stabilize the ATF4 protein. For this purpose, we knocked in a D933A mutation that inactivates its lipid kinase activity on top of the E545K mutant allele into the DLD1 *PIK3CA* mutant clone (Supplementary Fig. 5e,f). Figure 5g shows that the protein levels of both ATF4 and GPT2 were reduced in the double-mutant clones. As expected, AKT phosphorylation levels were also reduced in the double-mutant cells (Supplementary Fig. 5g). Importantly, the kinase inactivation mutation made the DLD1 *PIK3CA* E545K mutant clone less sensitive to glutamine deprivation (Fig. 5h). Moreover, knockdown of either PDK1 or RSK2 in both HCT116 and DLD1 *PIK3CA* mutant clones reduced ATF4 and GPT2 protein levels (Fig. 5i,j).

### Phosphorylation of ATF4 by RSK2 protects it from degradation

**Figure 6:**
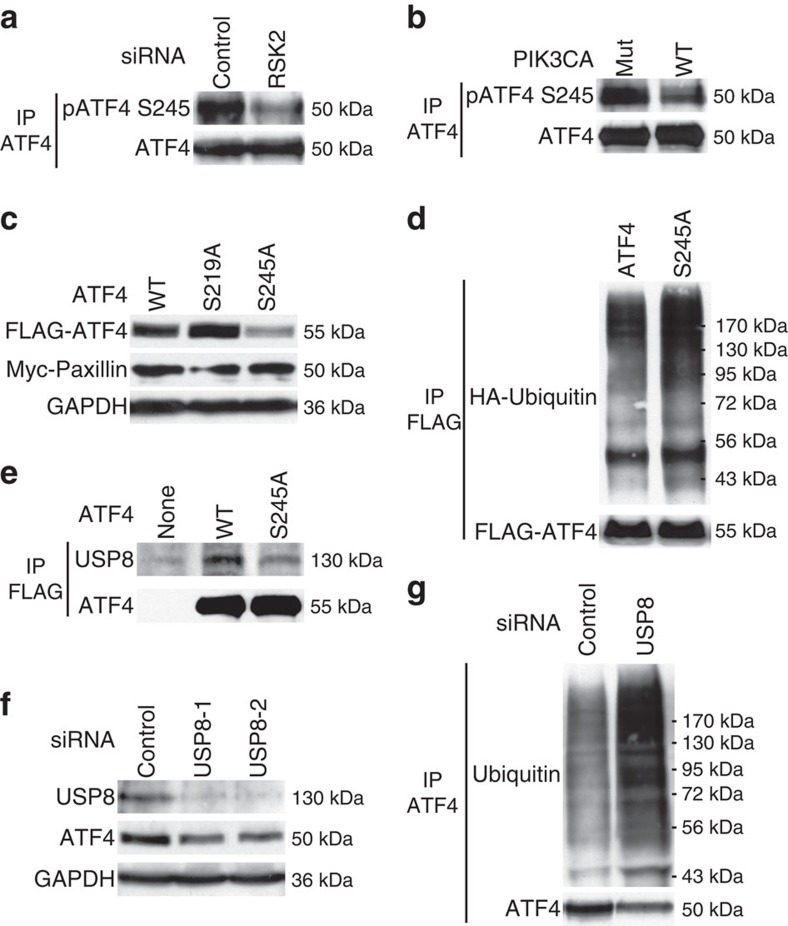
Phosphorylation of ATF4 S245 by RSK2 enhances its binding to USP8 and protects ATF4 from ubiquitin-mediated degradation. (**a**) Knockdown of RSK2 in a HCT116 *PIK3CA* mutant clone reduces pS245 ATF4 protein levels. HCT116 *PIK3CA* mutant cells were transfected with either control siRNA or siRNA against RSK2. Cell lysates were made 48 h post transfection, immunoprecipitated with anti-ATF4 antibodies and blotted with antibodies against either pS245 ATF4 or total ATF4. (**b**) Levels of pS245 ATF4 are higher in the HCT116 *PIK3CA* mutant clone than the WT clone. ATF4 proteins were immunoprecipitated and blotted with an anti-pS245 ATF4 antibody. (**c**) The ATF4 S245A mutant is less stable than the WT protein. The indicated constructs were expressed in the HCT116 *PIK3CA* mutant clone and cell lysates were blotted with the indicated antibodies. A Myc-paxillin construct was co-transfected as an internal control. (**d**) Ubiquitination levels of ATF4 S245A mutant are higher than that of WT protein. Constructs expression FLAG-tagged WT or S245A ATF4 were co-transfected with HA-ubiquitin into HCT116 *PIK3CA* mutant cells. Seventy-two hours post transfection, FLAG-tagged ATF4 proteins were immunoprecipitated and blotted with anti-human influenza hemagglutinin (HA) antibodies to detect ubiquitin. (**e**) The ATF4 S245A mutant binds to less USP8 than the WT protein. The indicated ATF4 constructs were transfected into HCT116 *PIK3CA* mutant cells. ATF4 immunocomplexes were pulled down by anti-FLAG antibodies and blotted with an anti-USP8 antibody. (**f**) Knockdown of USP8 with two different siRNAs in the HCT116 *PIK3CA* mutant clone reduces ATF4 protein levels. (**g**) Knockdown of USP8 increases the levels of ATF4 ubiquitination. Control siRNA or siRNA against USP8 was transfected into HCT116 *PIK3CA* mutant cells. Seventy-two hours post transfection, cells were treated with 5 μM MG132 for 6 h. Cell lysates were immunoprecipitated with anti-ATF4 antibodies and blotted with an anti-ubiquitin antibody.

RSK2 is a serine/threonine kinase that phosphorylates ATF4 at the serine 245 residue (S245)^26^. Knockdown of RSK2 in HCT116 *PIK3CA* mutant clone reduced levels of pS245 ATF4 (Fig. 6a). Therefore, we hypothesized that phosphorylation of ATF4 at S245 by the mutant p110α–PDK1–RSK2 signalling axis stabilizes ATF4. To test this hypothesis, we first examined ATF4 S245 phosphorylation in the HCT116 *PIK3CA* WT and mutant clones. As expected, levels of pS245 ATF4 were higher in the mutant clone than in the WT clone (Fig. 6b). Second, compared with the expression of a WT ATF4 construct, the expression of an unphosphorylatable ATF4 S245A mutant construct in the HCT116 *PIK3CA* mutant clone resulted in less ATF4 protein expression (Fig. 6c). In contrast, the ATF4 S219A mutant that abolishes the binding of ATF4 to β-TrCP1, an ubiquitin E3 ligase of ATF4 (ref. 24), generated more protein than the WT ATF4 (Fig. 6c). In agreement with our hypothesis that phosphorylation of ATF4 at the S245 residue stabilizes it, the ubiquitination levels of the ATF4 S245A mutant were higher than that of the WT protein (Fig. 6d). These data led us to postulate that phosphorylation of ATF4 S245 by RSK2 either reduces its binding affinity to an ubiquitin E3 ligase or recruits a deubiquitinase, thereby protecting ATF4 from degradation. To this end, we first tested the binding of WT and S245A mutant ATF4 to β-TrCP1. However, both WT and the mutant ATF4 bound to a similar amount of β-TrCP1 (Supplementary Fig. 6a). We then turned our attention to deubiquitinases and tested the binding of the WT ATF4 and S245A mutant to eight ubiquitin specific peptidases (USPs) (USP1, USP2, USP7, USP8, USP9X, USP10, USP14 and USP18). Among the USPs tested, only USP8 exhibited differential binding to WT versus mutant ATF4 (Fig. 6e and Supplementary Fig. 6b). In agreement with our hypothesis, the ATF4 S245A mutant bound to less USP8 than WT ATF4 (Fig. 6e). Knockdown of USP8 by two independent siRNAs in the HCT116 *PIK3CA* mutant clone resulted in reduced ATF4 protein levels (Fig. 6f) and increased ATF4 ubiquitination (Fig. 6g). However, knockdown of USP8 failed to make the PIK3CA mutant cells less sensitive to glutamine deprivation (Supplementary Fig. 6c), which might be due to additional roles of USP8 in regulating anti-apoptotic protein(s). Consistently, knockdown of USP8 significantly increased apoptosis of *PIK3CA* mutant cells even in the presence of glutamine (Supplementary Fig. 6c).

### TCA cycle metabolites are higher in *PIK3CA* mutant clones

**Figure 7:**
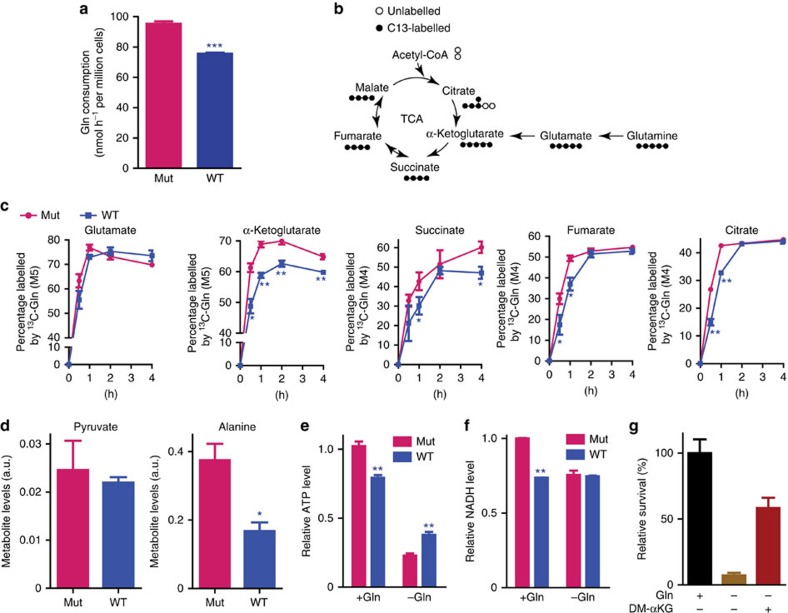
Mutation of *PIK3CA* increases glutamine consumption and conversion to α-KG. (**a**) Glutamine consumption rate of HCT116 *PIK3CA* mutant and WT clones. Cells were cultured with 2 mM initial concentration of [^13^C_5_]glutamine for 24 h. Concentration of leftover [^13^C_5_]glutamine were measured by gas chromatography mass spectrometry (GC-MS) to calculate glutamine consumption rate. (**b**) Schematic of TCA cycle and entry points for glutamine-derived carbons. (**c**) The flux of glutamine-derived TCA cycle intermediates is higher in HCT116 *PIK3CA* mutant cells than in the isogenic WT cells. Cells were cultured in the presence of 2 mM [^13^C_5_]glutamine, and direct glutamine-derived metabolites are measured by GC-MS at the indicated time points. Percentages of each labelled metabolite in total pool over the time course were plotted. (**d**) Intracellular pyruvate and alanine levels are higher in HCT116 *PIK3CA* mutant cells than in the isogenic WT cells. Total amounts of the indicated metabolite (unlabelled) were measured by GC-MS and then normalized to internal standards and cell numbers. (**e**,**f**) Relative levels of ATP and NADH in the HCT116 *PIK3CA* WT and mutant clones with or without glutamine. ATP (**f**); NADH (**g**). (**g**) α-KG rescues HCT116 mutant clone from cell death caused by glutamine (Gln) deprivation. Cells were grown in medium with the presence of the indicated nutrient for 72 h. Viable cell numbers were counted. Data are presented as mean±s.e.m. of three independent cultures. *P<0.05; **P<0.01; *t*-test.

As shown in Fig. 7a and Supplementary Fig. 7a, both the HCT116 and DLD1 mutant clones consumed more glutamine than their WT counter parts. Glutamine is converted to α-KG to replenish the TCA cycle (Fig. 7b). Because GPT2, an enzyme that converts glutamate to α-KG, is upregulated in *PIK3CA* mutant CRC cells, we measured glutamine flux in the paired isogenic lines by tracing [^13^C_5_]-glutamine over time. As shown in Fig. 7c, the flux of glutamine-derived TCA cycle intermediates including α-KG, succinate, fumarate and citrate was significant higher in the HCT116 *PIK3CA* mutant clone than in the WT clone at least at the early time points. Similar results were observed with the isogenic clones derived from DLD1 (Supplementary Fig. 7b). Although the flux of glutamine-derived glutamate was higher in DLD1 *PIK3CA* mutant cells than in the WT cells, the flux of glutamine-derived glutamate was similar in the HCT116 isogenic pair (Fig. 7c and Supplementary Fig. 7b). Nevertheless, the ratios of α-KG/glutamate were higher in the *PIK3CA* mutant clones than in the WT counterparts (Supplementary Fig. 7c). Similarly, compared with the isogenic *PIK3CA* WT clones, the pools of alanine and ratios of alanine/pyruvate were higher in the mutant counterparts (Fig. 7d and Supplementary Fig. 7d,e). These results support the premise that upregulation of GPT2 by oncogenic *PIK3CA* mutations leads to conversion of more α-KG from glutamine to replenish the TCA cycle. Consistently, knockdown of GPT2 in HCT116 *PIK3CA* mutant cells reduced levels of α-KG and TCA intermediates (for example, fumarate) that were derived from glutamine (Supplementary Fig. 7f,g). Conversely, overexpression of WT GPT2, but not enzymatically inactive mutant GPT2, increased glutamine-derived α-KG and TCA intermediates (Supplementary Fig. 7h,i).

A major product of the forward TCA cycle is NADH, which couples with oxidative phosphorylation to generate ATP. We therefore measured the amounts of ATP and NADH in the *PIK3CA* WT and mutant clones. In the presence of glutamine, the amounts of ATP and NADH were significantly higher in both the HCT116 and DLD1 mutant clones than in their WT counterparts (Fig. 7e,f and Supplementary Fig. 7j,k). The ratios of NADH/NAD were also significantly higher in the mutant clones than in the WT clones (Supplementary Fig. 7m). Although not statistically significant, the ATP/ADP ratios trended higher in the mutant clones (Supplementary Fig. 7l). However, under glutamine deprivation, the amount of ATP and the ATP/ADP ratio were significantly lower in the mutant clones than in the WT clones (Fig. 7e and Supplementary Fig. 7j,l), whereas the amounts of NADH and the ratios of NADH/NAD were similar in the mutant and WT clones (Fig. 7f and Supplementary Fig. 7k,m).

### α-KG rescues survival of Gln-deprived *PIK3CA* mutant cells

The results described above suggested that the generation of α-KG from glutamine to replenish the TCA cycle was critical to the survival of the *PIK3CA* mutant cells. To test this suggestion, we deprived the HCT116 *PIK3CA* mutant cell of glutamine and then supplemented the cells with 4 mM dimethyl-α-KG, a cell permeable form of α-KG. When deprived of glutamine, <8% cells survived after 3 days (Fig. 7g). In contrast, the addition of dimethyl-α-KG increased cell survival to ∼60%.

## Discussion

Our data demonstrate that oncogenic *PIK3CA* mutations reprogram glutamine metabolism through the upregulation of GPT2 in CRCs. Although a previous study by Weinberg and colleagues suggests that GPT2 is involved in oncogenic KRAS mutation-mediated glutamine metabolism in HCT116 cells, they did not investigate how K-ras regulates GPT2 (ref. 28). Importantly, using isogenic cell lines with either mutant allele or WT allele knockout, we clearly demonstrated that *KRAS* mutation does not make HCT116 and DLD1 CRC cells more dependent on glutamine. Moreover, both SW480 and LOVO CRC cell lines harbour oncogenic *KRAS* mutations, but the two cell lines were resistant to glutamine deprivation. Thus, our data suggest that mutant *KRAS* is not a key determinant of glutamine dependency in CRCs. Consistently, knock-in of mutant K-ras in mouse and a human cell line actually reduces or does not affect PI3K/AKT activities^29^,^30^, although WT K-ras interacts and activates PI3K (ref. 31). In contrast, our data provide compelling evidence that oncogenic *PIK3CA* mutations in CRCs render them more sensitive to glutamine deprivation. Given that several studies show that PI3K/AKT signalling regulates glucose metabolism^32^,^33^, it is surprising to observe that HCT116 and DLD1 *PIK3CA* mutant and WT knockout clones could grow without glucose. Our data could be interpreted that upregulation of PCK2 in HCT116 and DLD1 cells deprived of glucose attenuates glucose dependency of these cells.

Our data also show that CRC cells harbouring *PIK3CA* mutations, but not those cells with WT *PIK3CA*, are sensitive to growth inhibition by AOA, a compound that blocks the conversion of glutamate to α-KG. These findings constitute a proof of principle that targeting glutamine metabolism could be a useful approach to treating CRCs harbouring P*IK3CA* mutations. Since the first *PIK3CA* mutations in human cancers were discovered^14^, huge efforts have been made to develop small molecule inhibitors of p110α (ref. 34). However, because the catalytic domains of class I lipid kinases, p110α, p110β, p110γ and p110δ, are highly conserved, it is difficult to design small molecules that specifically inhibit the p110α isoform^35^, and pan-PI3K inhibitors are highly toxic. Moreover, the p110α inhibitors developed to date do not discriminate between mutant and WT p110α proteins. A recent study showed that cancer cells quickly develop resistance to a p110α-specific inhibitor BYL719 (ref. 36). Our study suggests that targeting glutamine metabolism may provide a specific form of therapy for cancer patients harbouring *PIK3CA* mutations.

The findings reported here demonstrate that GPT2 is the key determinant of glutamine sensitivity in *PIK3CA* mutant CRC cells. GPT2 is an aminotransaminase that converts glutamate to α-KG, which is a TCA cycle intermediate. Metabolic profiling shows that amounts of α-KG are significantly higher in the *PIK3CA* mutant clones than in the WT clones and that the other TCA cycle intermediates are also higher in the mutant clones than in the WT clones. Moreover, α-KG largely rescues *PIK3CA* mutant cells from cell death caused by glutamine deprivation, suggesting that α-KG is a key metabolite required for *PIK3CA* mutant cell growth. Together, these data suggest that upregulation of GPT2 by *PIK3CA* mutations produces more α-KG from glutamine to replenish the TCA, thereby generating more ATP and intermediates for macromolecule synthesis to sustain rapid growth of *PIK3CA* mutant tumours. This is in agreement with our observation that both the ATP concentration and the ATP/ADP ratios were higher in the *PIK3CA* mutant cells than in the WT cells.

AOA potently inhibits xenograft tumour growth of CRCs with *PIK3CA* mutations, despite its low potency to inhibit growth of CRC cells in culture. A similar discrepancy was also observed with a breast cancer cell line^23^. We postulate that tumour cells are more dependent on glutamine in the *in vivo* microenvironment than the *in vitro* tissue culture conditions. Although AOA is a pan-aminotransferase inhibitor, our data clearly demonstrate that the tumour inhibitory effect of AOA on *PIK3CA* mutant xenografts is indeed on-target to GPT2, because GPT2 knockdown cells grow much slower in nude nice and these tumours are insensitive to AOA treatment. Consistently, AOA treatment reduces amounts of α-KG and alanine, but increases amount of pyruvate and glutamate, in *PIK3CA* mutant xenograft tumours. Currently, only pan-aminotransferase inhibitors are available. It is conceivable that a GPT2-specific inhibitor could be more potent and less toxic. Given that GPTs are also involved in gluconeogenesis in liver and kidney, GPT inhibitors may cause hypoglycaemia. However, a study by Lindblom *et al.* shows that GPT1, but not GPT2, is predominantly expressed in liver and kidney^37^. Thus, GPT2-specific inhibitors could still have a favourable therapeutic index.

It is generally believed that AKTs are the key mediators of the oncogenic signalling of PI3Ks (ref. 38). This study, however, revealed a novel p110α–PDK1–RSK2–ATF4–GPT2 pathway that regulates glutamine metabolism. We demonstrated that blocking this pathway inhibits PIK3CA mutant tumour growth *in vitro* and *in vivo*, suggesting that this novel signalling pathway also plays a critical role in tumorigenesis driven by *PIK3CA* mutations. While our previous studies showed that AKT signalling drives metastasis of *PIK3CA* mutant tumours^39^, here we propose the p110α–PDK1–RSK2–ATF4–GPT2 signaling axis reprogrammes glutamine metabolism that is required to generate energy and metabolites to sustain rapid growth in *PIK3CA* mutant tumours.

In summary, we have discovered a novel mechanism through which mutant p110α perturbs the regulation of glutamine and makes cancer cells more dependent on adequate glutamine levels. We demonstrated that mutant p110α activates RSK2 kinase through PDK1. Activated RSK2 then phosphorylates ATF4 at the serine residue 245, which in turn recruits the deubiquitinase USP8 and protects ATF4 from ubiquitin-mediated degradation (Supplementary Fig. 8). This pathway can be targeted by various means, which may provide potential selective therapies for the treatment of the large number of patients whose tumours harbour *PIK3CA* mutations. Future experiments may extend our study to a large panel of CRC cell lines, patient-derived xenografts and transgenic *PIK3CA* mutant mouse models.

## Methods

### Cell culture

CRC cell lines HCT116, DLD1, RKO, HT29, SW480 and LOVO were purchased from American Type Culture Collection. Isogenic *PIK3CA* mutant and WT and *KRAS* mutant, and WT HCT116 and DLD1 cell lines were generated previously^17^,^20^. All cell lines were maintained in McCoy's 5A medium (Fisher Scientific, Catalogue No. SH30200) containing 10% fetal bovine serum (FBS, Fisher Scientific, Catalogue No. SH30910). Tissue cultures were routinely assayed for mycoplasma to ensure that they were mycoplasma-free. For glutamine deprivation, cells were cultured in glutamine- and pyruvate-free DMEM with 4.5 g l^−1^ glucose (Fisher Scientific, Catalogue No. SH30081) with 10% dialysed FBS (dFBS; Invitrogen, Catalogue No. 26400). For glucose deprivation, cells were cultured in glucose-free DMEM (Invitrogen, Catalogue No. A14430) with 10% of dFBS.

### Reagents

L-Glutamine, L-glutamine-^13^C_5_, D-glucose, dimethyl α-KG and AOA were purchased from Sigma. Inhibitors LY294002 (pan-PI3K inhibitor), BYL-719 (p110α-specific inhibitor), BEZ235 (PI3K/mTOR dual-specificity inhibitor), GSK2334470 (PDK1 inhibitor), GSK690693 (AKT inhibitor), CHIR-99021 (GSK3β inhibitor), rapamycin (mTOR inhibitor), BI-D1870 (pan-RSK inhibitor), FMK (RSK2 inhibitor) and MG132 (proteasome inhibitor) were purchased from Selleck Chemicals. siRNAs for ATF4 (SI03019345 and SI04236337) and PDK1 (SI00301140 and SI00301154) were purchased from Qiagen. siRNAs for RSK2 (J-003026-10 and J-003026-12) were purchased from Dharmacon. siRNAs for USP8 (SR306014A and SR306014B) were purchased from Origene. shRNAs for GPT2 (TRCN0000035025 and TRCN0000035026) were purchased from Sigma. Antibodies used in this study are listed in Supplementary Table 1.

### Plasmid construction

A GPT2 cDNA clone was purchased from Open Biosystems. The GPT2 ORF was subcloned into the pCMV-3Tag1A vector with HindIII and SalI. Then the FLAG-GPT2 sequence was PCRed out and subcloned into pCDNA3.1zeo with KpnI and XbaI. An enzymatically inactive form of GPT2 (K341H) was generated with the Quick-change Site-Directed Mutagenesis kit (Agilent Technologies). ATF4 and β-TrCP plasmids were obtained from Addgene. A FLAG-tagged ATF4 expression vector was constructed by subcloning ATF4 ORF into the pCMV-3Tag1A vector with BamHI and XhoI. ATF4 S219A and S245A mutations were made with the Quick-change Site-Directed Mutagenesis kit (Agilent Technologies). Then the pCMV-ATF4 S219A construct was digested with NotI and XhoI, and the ATF4 S219A fragment was subcloned into pCDNA3.1zeo. To make the construct expressing sgRNA to knock out GPT2, the CRISPR/Cas9 vector pX330 was digested with BbsI and ligated with annealed oligonucleotides. All primers used in this study are listed in Supplementary Table 2.

### Plasmid transfection

Plasmids were transfected into cells with Lipofectamine 3000 (Invitrogen) according to the manufacturer's instructions. For transient expression, cells were lysed 72 h after transfection. For stable expression (FLAG-GPT2 expression), cells were selected with 0.1 mg ml^−1^ Zeocin (Invitrogen) for 7 days.

### Serial analysis of gene expression

SAGE libraries were generated from 5 μg of total RNA, with reagents from the NlaIII Digital Gene Expression-Tag Profiling preparation kit (Illumina, San Diego, CA) according to the manufacturer's instructions. This protocol results in a SAGE library wherein each tag is surrounded by adaptors suited for sequencing on the Illumina Genome Analyzer platform (Illumina). Each library was loaded on one channel of an Illumina flow cell, and 17 cycles of sequencing were performed. Sequence tags with Phred-equivalent score >20 for every base were used for analysis.

### RT–PCR analysis

RNAs were extracted using RNeasy mini kits (Qiagen) according to the manufacturer's instructions. A unit of 1 μg total RNA was used for reverse transcription using the Superscript First-Strand kit (Invitrogen) and cDNAs were used as templates for PCR. Primers are listed in Supplementary Table 2.

### Real-time PCR analysis

RNAs from frozen tumours were extracted as described above. The Taqman assay system was used for qRT–PCR using GPT2 probes (Hs00370287, Applied Biosystems) with IQ super mix (Catalogue No. 170-8860, Bio-Rad). Expression levels of GPT2 in each tumour was normalized to that of β-2-microglobulin. The PIK3CA mutation status of the human CRC specimens is listed in Supplementary Table 3.

### Mining TCGA data

For TCGA colon adenocarcinoma and rectal adenocarcinoma patients, RNA-seq files in the *rsem.genes.normalized_results format were downloaded from the TCGA website for all tumours and, where available, matched normal tissue samples. This file format provides normalized fragments per kilobase of exon per million reads mapped (FPKM) values, using the RSEM software^40^, for each gene. In addition, the somatic mutation.maf files were downloaded from the same site for each tumour type. For the PIK3CA mutant group, tumours with the following characteristics were excluded: (1) nonsense mutations that generate stop codons; (2) unknown mutations that were not confirmed; and (3) possible tumour–normal mismatches that had multiple mutations in PIK3CA.

### Gene targeting

GPT2 knockout clones were generated with CRISPR/Cas 9. A pX330 plasmid expressing sgRNA that targets exon 4 of GPT2 genomic sequences was transfected into HCT116 cells, and single clones were selected. Knockout clones were verified by sequencing and western blot analysis.

The PIK3CA D933A targeting vector was constructed with the USER system, and targeted cells were generated with rAAV as described previously^41^,^42^. Briefly, vector arms were created by PCR from genomic DNA using *HiFi* Taq (Invitrogen) and validated by sequencing before viral production and infection. Stable G418-resistent clones were then selected for PCR screening as reported^42^. Targeted clones were genotyped by RT–PCR and sequencing. Primers for constructing the targeting vector and screening for knock-in clones are listed in Supplementary Table 3.

### Immunoblotting and immunoprecipitation

Cells were lysed in RIPA buffer (10 mM Tris (pH 7.4), 150 mM NaCl, 5 mM EDTA (pH 8.0), 0.1% SDS, 1% Triton-X100, 1 mM dithiothreitol, 1 mM phenylmethyl sulphonyl fluoride and complete Protease Inhibitor Cocktail tablet (Roche); supplemented with phosphatase inhibitors (1 mM Na_3_VO_4_, 50 mM NaF, 1 mM β-glycerophosphate and 20 mM sodium pyrophosphate)). Lysates were cleared by centrifugation at 14,000 r.p.m. for 10 min, and protein concentration in supernatants was determined by the BCA protein assay kit (Pierce). Equal amounts of total protein were used for immunoblotting. For immunoprecipitation, cells were lysed as described above. Cleared cell lysates were incubated with antibody for 1 h, and then protein A and/or protein G for 1 h. Protein A/G beads were washed with lysis buffer three times, and then boiled with SDS-loading buffer followed by immunoblotting. Original images of western blots are shown in Supplementary Fig. 9.

### Luciferase reporter assays

The 1.5-kb promoter of GPT2 was subcloned into the pGL3 vector (Promega) to obtain a pGL3-GPT2 promoter-LUC plasmid. pGL3-GPT2 promoter-LUC was co-transfect with pCMV-ATF4 and an internal control β-galactosidase expressing pCH110 plasmid (abbreviated as pCH110 β-gal) or *Renilla* luciferase expressing pRL (Promega). Forty-eight hours after transfection, cells were collected for Luciferase assay according to the manufacturer's instructions (Promega). Luminescence was measured with an EnVision 2103 Multilabel Plate Reader (PerkinElmer). β-galactosidase activity was measured with a β-Gal assay kit (Invitrogen). Relative luciferase activity was expressed as the ratio of luminescence normalized to the internal control. Two putative ATF4-binding sites in the GPT2 promoter were predicted by TFseach v1.3. Mutations in the GPT2 promoter were generated with the Quickchange kit (Agilent Technologies) and primers in Supplementary Table 3. The uORFATF4 plasmid was a kind gift from Dr Ron Wek at Indiana University.

### Cell proliferation assays

Cells were plated in 96-well plates at 2,000 cells per well, 24-well plates at 1 × 10^4^ cells per well and 6-well plates at 2 × 10^5^ cells per well in complete DMEM (20 mM glucose, 2 mM glutamine, 10% dFBS; Invitrogen). After 24 h, cells were washed with PBS, and changed to either glutamine-free DMEM (with 20 mM glucose) or glucose-free DMEM (with 2 mM glutamine) containing 10% dFBS. Cells (including floating cells in medium) were collected and counted by Trypan-Blue exclusive assay. Cell numbers in 96-well plates were measured by Cell Counting Kit 8 (Dojindo) according to the manufacturer's instructions.

### Flow cytometry

Cells were fixed with methanol and then incubated at 37 °C for 30 min in 5% normal goat serum diluted in PBS. Propidium iodide solution was used to stain cells at 4 °C for 1 h. Cells were analyzed on an Epics XL flow cytometer. WinMDI2.9 software was used for data analysis. Cell debris and aggregates were excluded by propidium iodide gating. Percentages of sub-G1, G1, S and G2/M populations were determined by histograms generated by WinDI2.9.

### Gene silencing

Plasmids expressing shRNAs were transfected into cells. Two days post transfection, cells were selected with 1 μg ml^−1^ puromycin for 7 days. Puromycin-resistant cells were pooled, amplified and analysed. For genes silenced by siRNAs, siRNAs were transfected into cells with Lipofectamine 3000. Three days post transfection, cells were collected for further analyses.

### Ubiquitination assays

Cells were pretreated with 5 μM of MG132 for 6 h as previously described^43^, and cell lysates were immunoprecipitated with antibodies against either ATF4 or FLAG. Beads were washed three times with washing buffer (10 mM Tris (pH 7.4), 1 M NaCl, 1 mM EDTA (pH 8.0), 1% NP-40 and 0.2% SDS). The immunocomplexes were resolved in SDS–PAGE gels for western blot analyses.

### Polysome profile analysis

Three tumours (∼250 mm^3^ size) of each genotype were snap-frozen in liquid nitrogen, pulverized and then lysed in 1 ml of lysis buffer (50 mM HEPES-KOH (pH 7.4), 5 mM MgCl_2_, 250 mM KCl, 2% Triton X-100, 8.5% sucrose, 100 μg ml^−1^ cycloheximide, 1 mM dithiothreitol, 200 U ml^−1^ RNase inhibitor (RNaseOUT, Invitrogen), EDTA-free protease inhibitor (Roche Applied Science) and 10 mM ribonucleoside vanadyl complex (New England Biolabs)), kept on ice for 20 min and then passed 15 times through a 23-gauge needle. Lysates were spun at 14,000 r.p.m. for 15 min, and supernatants were collected. Approximately 10–15 absorbance units (260 nm) of lysates were layered over cold 10–50% sucrose gradients in buffer (50 mM HEPES-KOH (pH 7.4), 5 mM MgCl_2_ and 250 mM KCl). Gradients were centrifuged at 17,000 r.p.m. in a Beckman SW28 rotor for 15 h at 4 °C. After centrifugation, 12 fractions (1.2 ml per fraction) were collected. RNA from each fraction was isolated using TRIzol LS reagent (Invitrogen), and an equal volume from each fraction was used for cDNA synthesis. The relative quantities of specific mRNAs were measured by qRT–PCR.

### Inhibitor treatments

To assay ATF4 protein stability, HCT116 PIK3CA mutant or WT cells were cultured in complete McCoy5A medium and treated with vehicle or inhibitors for 12 h, then lysed for western blot analyses. For ubiquitination assay by immunoprecipitation, cells were treated with 5 μM MG132 for 6 h before they were lysed. For long-term treatment with MG132, cells were exposed to 0.2 μM MG132 for 48 h. Cells were lysed for western blot analysis, or fixed for flow cytometry.

### Xenograft studies

Animal experiments were approved by the Case Western Reserve University Institutional Animal Care and Use Committee. As described in ref. 44, 3 million cells were injected subcutaneously into the flanks of 4- to 6-week-old female athymic nude mice. Mice were randomly assigned into treatment groups (5 mice per group). When average tumour volume reached about 100 mm^3^, mice were injected intraperitoneally with vehicle or with 5 mg kg^−1^ or 10 mg kg^−1^ of AOA every day. Tumour volumes were measured with an electronic caliper and calculated as length × width^2^/2. Body weights were measured at the beginning and at the end of treatment. These experiments were not blinded.

### Glutamine consumption

One million cells were plated in complete medium in a T25 flask. When the cells were ∼70% confluent, they were washed twice with PBS, and changed into medium containing 2 mM of [^13^C_5_]L-glutamine (C_Gln0_) and cultured for 24 h. Medium was collected and frozen at −80 °C. To measure the glutamine concentration remaining in the medium (C_Gln___end_), 50 μl of medium was mixed with 50 μl of 2 mM [^12^C]L-glutamine as internal standard and 500 μl of pre-chilled (−80 °C) methanol was added. The supernatants were dried with nitrogen gas. TBDMCS (tert-butyldimethylchlorosilane, REGIS Technologies):acetonitrile (2:1) was used for derivatization of metabolites at 60 °C for 1 h. Samples were injected into GC-MS (Agilent Technologies) for analysis. Glutamine concentration in medium was determined by normalization to the internal standard. Glutamine (Gln) consumption rate=(C_Gln0_−C_Gln___end_) × medium volume/cell numbers/incubation time.

### Metabolic assays and stable isotope tracing

A million cells were plated in each T25 flask. When cells reached ∼70% confluency, they were washed with PBS twice and changed to medium containing 2 mM of [^13^C_5_-]glutamine. Cells were collected with 1 ml pre-chilled (−80 °C) methanol at 0.5, 1, 2 and 4 h. A unit of 5 μM of heptadecanoic acid, 2.5 μM of [3,3,4,5,5,5-^2^H6]4-hydroxypentanoate and 2.5 μM of [2,2,3,3,4,4,5,5,6,6,7,7,7-^2^H13] heptanoate were added as internal standards. Metabolites were extracted by homogenization and sonication on ice. Cell debris was removed by centrifugation at 14,000 r.p.m. for15 min at 4 °C. The supernatant was dried with nitrogen gas. TBDMCS:acetonitrile (2:1) was used for derivatization of metabolites at 60 °C for 1 h. Samples (1 μl) were injected into GC-MS (Agilent Technologies) for metabolite profiling. The direct [^13^C_5_-]glutamine metabolites are ^13^C_5_-glutamate (M5 glutamate), ^13^C_5_-α-KG (M5 α-KG), ^13^C_4_-succinate (M4 succinate), ^13^C_4_-fumarate (M4 fumarate) and ^13^C_4_-citrate (M4 citrate). For metabolite flux analyses, the total pool of each metabolite was considered as 100%. Metabolite fluxes were presented as percentage of direct glutamine-derived metabolites in the total pool. For analysis of metabolite levels, the amounts of labelled glutamate, α-KG and TCA intermediates at 2 h were measured and normalized to internal standards. Unlabelled pyruvate and alanine were measured and normalized to D13 heptanoate.

### Assays of ATP/ADP and NADH/NAD

The amounts of ATP and the ATP/ADP ratios were measured with an ADP/ATP ratio assay kit (Abcam) according to the manufacturer's instructions. The amounts of ATP were determined by an ATP standard curve, and normalized to the protein concentrations. The amounts of NADH and NADH/NAD ratios were measured with an NAD/NADH colorimetric kit (BioVision). The NADH concentrations were determined by a NADH standard curve, and normalized to the protein concentrations.

### Statistical analyses

We applied the *t*-test to compare the means between two groups, assuming unequal variances. For xenograft growth, analysis of variance was performed for repeated measurements to test whether there was an overall difference in the tumour size.

### Data availability

The TCGA data referenced during the study are available in a public repository from the TCGA website. The authors declare that all the other data supporting the findings of this study are available within the article and its Supplementary Information files and from the corresponding author on reasonable request.

## Additional information

**How to cite this article:** Hao, Y. *et al.* Oncogenic *PIK3CA* mutations reprogram glutamine metabolism in colorectal cancer. *Nat. Commun.* 7:11971 doi: 10.1038/ncomms11971 (2016).

## Supplementary Material

Supplementary Information Supplementary Figures 1-9 and Supplementary Tables 1-3

Supplementary Data 1 Gene expression analysis of isogenic PIK3CA WT and mutant only cells.
