# Supplementary material for: Oncogenic PIK3CA mutations reprogram glutamine metabolism in colorectal cancer
Source: Nat Commun. 2016 Jun 20;7:11971. doi: 10.1038/ncomms11971 (PMC4915131; doi:10.1038/ncomms11971)
Supplement: Supplementary Information — Supplementary Figures 1-9 and Supplementary Tables 1-3 [file ncomms11971-s1.pdf]

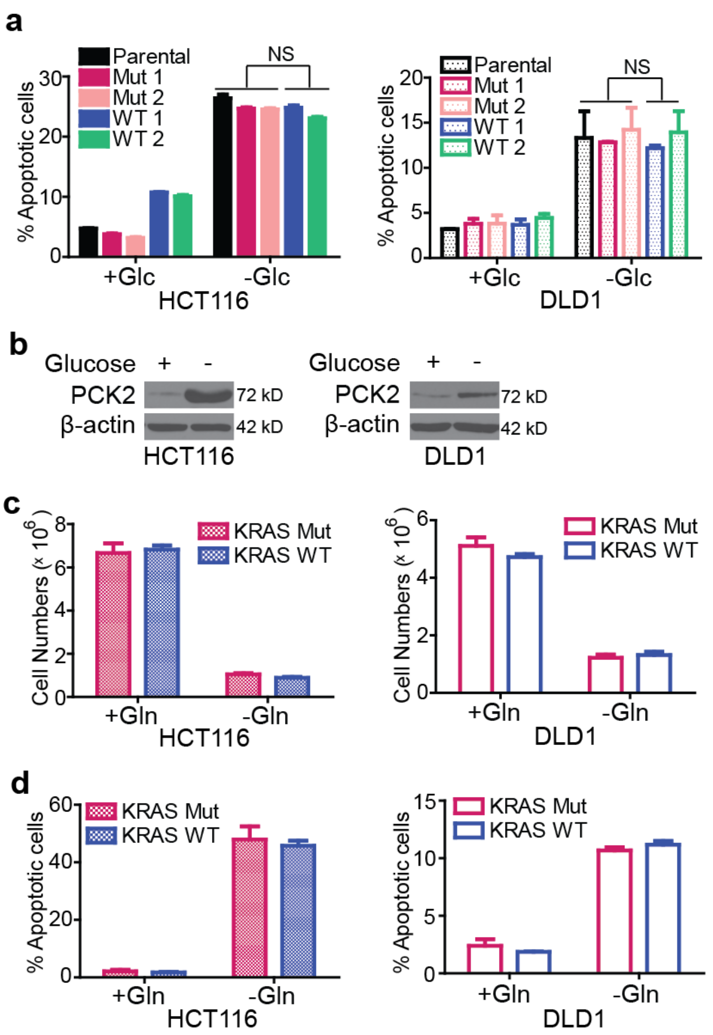

Supplementary Figure 1. PIK3CA mutations, but not KRAS mutations, render CRCs dependent on glutamine but not glucose.

(a) PIK3CA mutant and WT isogenic clones of HCT116 and DLD1 do not display differential sensitivity to glucose deprivation. Cells of the indicated genotypes were cultured with or without glucose (Glc) in medium with 10% dialyzed FBS for 72 hours. Apoptotic cells were quantified as sub-G1 populations.

(b) Glucose deprivation up-regulated protein levels of phosphoenolpyruvate carboxykinase 2 (PCK2). The indicated cells were grown in medium with or without glucose for 48 hours. Cell lysates were blotted with the indicated antibodies.

(c) & **(d)** KRAS mutations do not render CRCs more dependent on glutamine. The indicated CRC cell lines were grown with or without glutamine for 72 hours. Cell numbers were counted (c) and apoptotic cells were quantified (d).

Data are presented as mean ± SEM of three independent cultures. NS: not significant; t test.

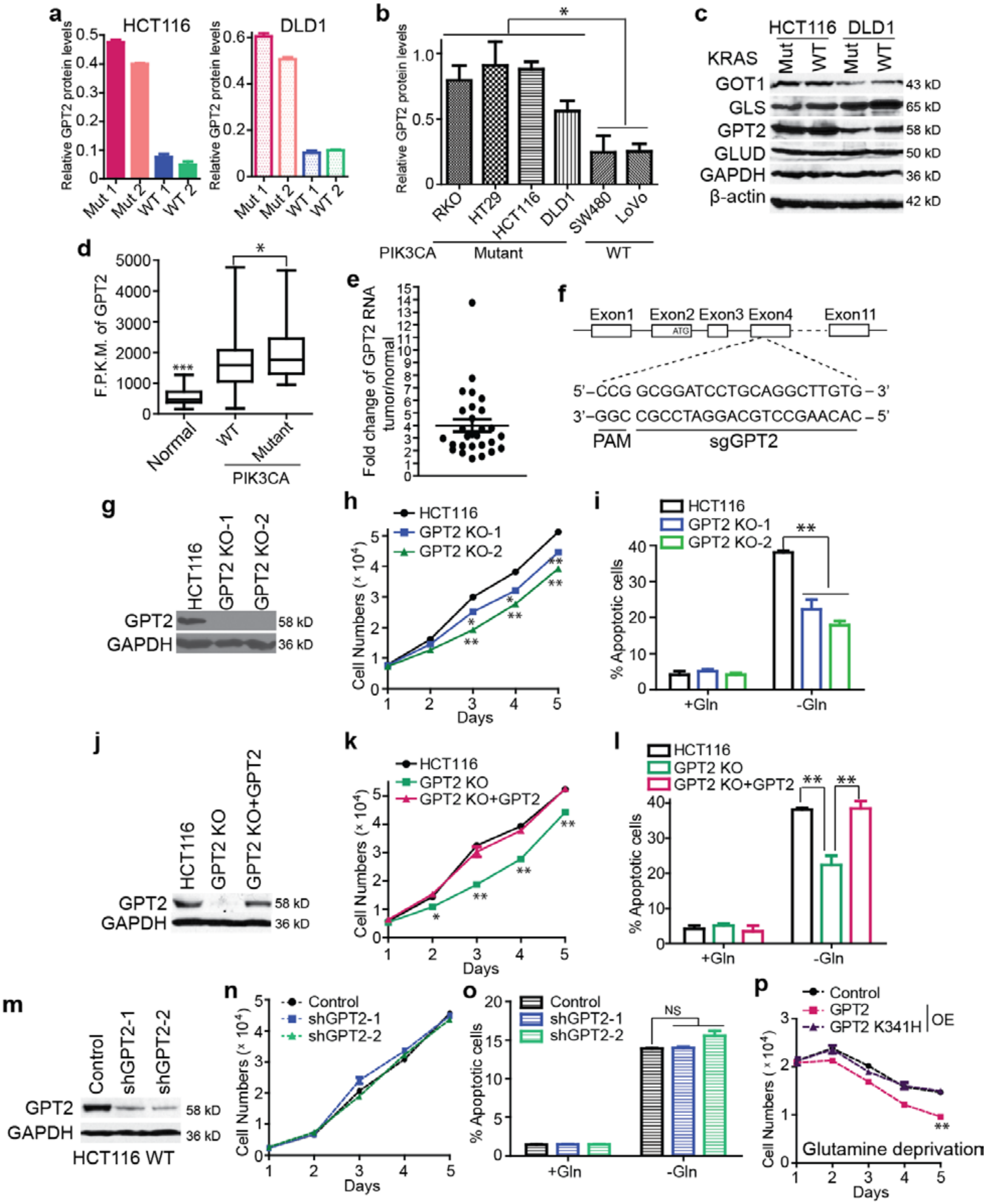

Supplementary Figure 2. Up-regulation of GPT2 by PIK3CA mutations leads to dependency on glutamine.

(a) GPT2 protein levels are higher in PIK3CA mutant clones. The intensities of GPT2 and GAPDH bands shown in Fig. 2b were quantified using Image J software. GPT2 protein levels were normalized to GAPDH.

(b) Quantification of Western blot analyses of three independent experiments of the indicated cell lines as shown in Figure 2d.

(c) KRAS mutations do not affect protein levels of enzymes involved in glutamine metabolism in CRCs. Cell lysates of the indicated genotype were blotted with the indicated antibodies.

(d) GPT2 expression levels are higher in PIK3CA mutant CRCs compared to CRCs with WT PIK3CA in the TCGA dataset. For TCGA colon adenocarcinoma (COAD) and rectal adenocarcinoma (READ) patients, RNA-seq files were downloaded from the TCGA website for all tumors and, where available, matched normal tissue samples. Additionally, the somatic files were downloaded from the same site for each tumor type. For the PIK3CA mutant group, tumors with the following characteristics were excluded: (1) nonsense mutations which generate stop codons in PIK3CA; (2) mutations that were not confirmed; and (3) tumors with multiple mutations in PIK3CA, indicating tumor/normal mismatch. Normal (n = 51), CRCs with WT PIK3CA (n=216) and CRCs with PIK3CA mutations or loss of PTEN (n= 57). FPKM: fragments per kilobase of exon per million reads mapped.

(e) GPT2 expression is up-regulated in CRCs. GPT2 RNA-seq expression data of CRCs and their matched normal tissues were extracted from the TCGA database. Fold changes of GPT2 for each tumor/normal pair are plotted (n=28).

(f) to (**i**) Knockout of GPT2 makes HCT116 cells grow slower but resistant to glutamine deprivation. Exon 4 of GPT2 was targeted by CRISPR/Cas9 genome editing technology. Guide RNA (sgRNA) sequences are shown (e). Western blot analysis of GPT2 protein in parental cells and two independently-derived GPT2 knockout clones (f). GPT2 knockout clones grow slower than parental cells. Cell proliferation was assayed by counting cell number for five consecutive days (g). Compared to parental cells, GPT2 knockout clones are more resistant to glutamine deprivation. Cells were grown with or without glutamine for 3 days and apoptotic cells were measured by FACS analysis of sub-G1 cell population (h).

(j) to (**l**) Reconstitution of GPT2 in GPT2 knockout cells makes them more sensitive to glutamine deprivation. GPT2 knockout clone 1 (KO-1) was transfected with a plasmid expressing GPT2. Stable pools were selected. Western blot shows re-expression of GPT2 (i). Reconstitution of GPT2 makes the knockout cells grow faster (j). Cells of the indicated genotypes were grown with or without glutamine for 72 hours. Apoptotic cells were quantified (k).

(m) to (**n**) Knockdown of GPT2 in a HCT116 PIK3CA WT clone has no impact on sensitivity to glutamine deprivation. HCT116 PIK3CA WT cells was transfected with control shRNA or two independent shRNA against GPT2. Stable pools were selected. Knockdown of GPT2 was confirmed by Western blots analysis (l). Cell proliferation was assayed by counting cell number for five consecutive days (m). Cells were grown with or without glutamine for 3 days and apoptotic cells were measured by FACS analysis of sub-G1 cell population (n).

(p) Overexpression (OE) of WT GPT2, but not inactive mutant, in the HCT116 PIK3CA WT clone makes them more sensitive to glutamine deprivation. Equal numbers of cells expressing WT or enzymatically inactive GPT2 were grown in medium without glutamine and cell numbers were counted for five consecutive days.

Data are presented as mean ± SEM of three independent cultures. * $p < 0.05$; ** $p < 0.01$; *** $p < 0.001$.

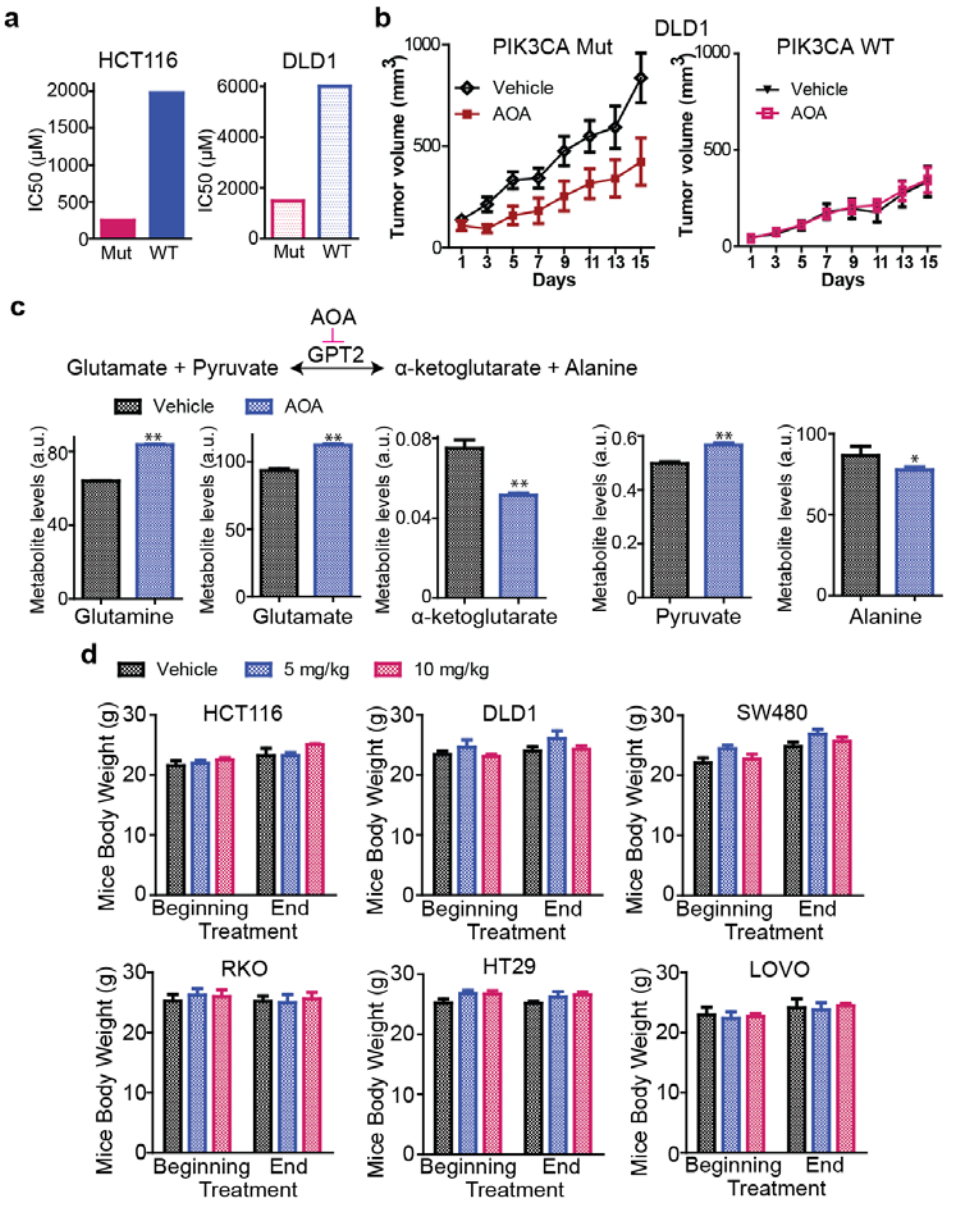

Supplementary Figure 3. AOA inhibits PIK3CA mutant tumor growth.

(a) IC_{50} of AOA in HCT116 and DLD1 PIK3CA WT and mutant clones.

(b) AOA inhibits xenograft growth by a DLD1 PIK3CA mutant clone (Mut 1) but not a PIK3CA WT clone (WT1). Three million cells were injected subcutaneously and bilaterally into athymic nude mice. Once tumors reached an average size of 100 mm^3, 10 mg/kg of AOA was injected IP once a day for two weeks.

(c) AOA treatment reduces amounts of α-ketoglutarate and alanine, but increases amounts of glutamine, glutamate and pyruvate, in tumors formed by HCT116 PIK3CA mutant cells. Xenograft tumors were treated with vehicle or 10 mg/kg of AOA daily for 2 weeks. Tumors were harvested and the indicated metabolites were measured by GC-MS. N = 4 tumors for each group. a.u.: arbitrary unit. * $p < 0.05$; ** $p < 0.01$; t test.

(d) Body weights of the mice with xenografts established from the indicated CRC cells before and after AOA treatment. N= 5 mice in each group.

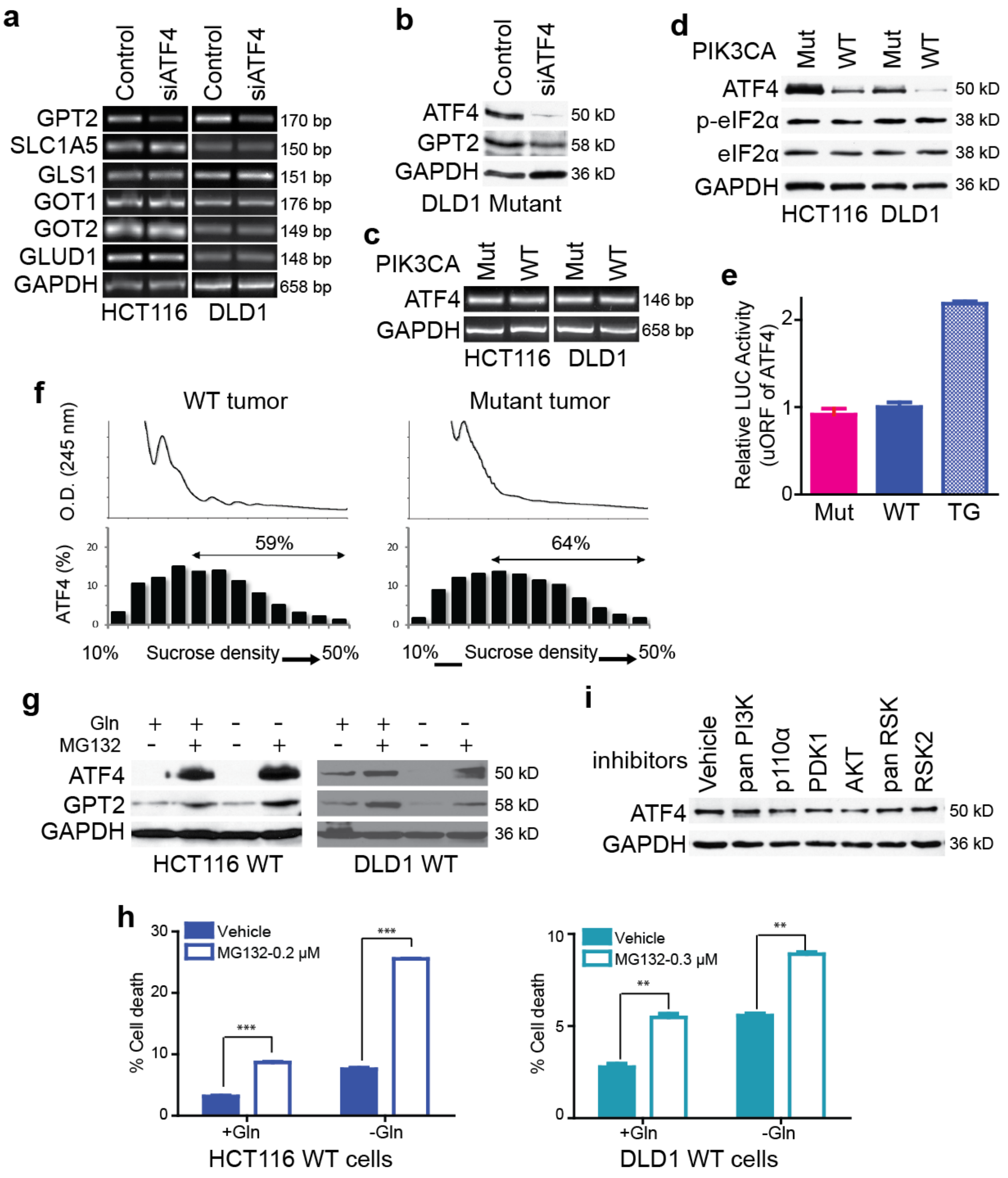

Supplementary Figure 4. ATF4 regulates GPT2 transcription.

(a) Knockdown of ATF4 reduces GPT2 mRNA levels in PIK3CA mutant clones. HCT116 and DLD1 PIK3CA mutant clones were transfected with either control siRNA or siRNA against ATF4. RT-PCRs were performed with the indicated genes.

(b) Knockdown of ATF4 reduces GTP2 protein levels. DLD1 PIK3CA mutant clones were transfected with either control siRNA or siRNA against ATF4. Cell lysates were blotted with the indicated antibodies.

(c) Expression levels of ATF4 mRNA are similar in PIK3CA WT and mutant clones. RT-PCR analyses of ATF4 and GAPDH in the indicated clones.

(d) Levels of p-eIF2α are similar between PIK3CA WT and mutant clones. Cell lysates of the indicated clones were blotted with the indicated antibodies.

(e) uORF-dependent translation of ATF4 is similar between PIK3CA WT and mutant clones. ATF4 uORF-luciferase plasmid and internal control plasmid expressing Renilla luciferase were co-transfected into HCT116 PIK3CA mutant or WT cells. Forty-eight hours post-transfection, cells were assayed for luciferase activity. Thapsigargin (TG), an ER stress inducer which activates ATF4 uORF, was used to treat cells for 3 hours as a positive control.

(f) ATF4 mRNA loading onto polysomes is similar in PIK3CA WT and mutant tumors.

(g) Treatment with proteasome inhibitor MG132 stabilizes ATF4 protein and up-regulates GPT2 levels in HCT116 and DLD1 PIK3CA WT cells. Cells were grown in the indicated conditions and treated with 0.2 µM of MG132 for 48 hours. Cell lysates were blotted with the indicated antibodies.

(h) MG132 treatment increases cell apoptosis. Cells of the indicated genotypes were grown with or without glutamine and treated with 0.2 µM of MG132 for 48 hours. Apoptotic cells were quantified.

(i) Inhibitors of PI3K, PDK1 and RSK2 do not affect ATF4 protein levels in a HCT116 PIK3CA WT clone. Inhibitors include 10 µM LY294002 (pan-PI3K inhibitor), 5 µM BYL-719 (p110α-specific inhibitor), 6 µM GSK2334470 (PDK1 inhibitor), 10 µM GSK690693 (AKT inhibitor), 10 µM BI-D1870 (pan-RSK inhibitor), and 10 µM FMK (RSK2 inhibitor). Cells were treated with vehicle or the indicated inhibitors for 12 hours and then lysed for Western blot analyses with the indicated antibodies.

Data are presented as mean ± SEM of three independent cultures. *** $p < 0.001$.

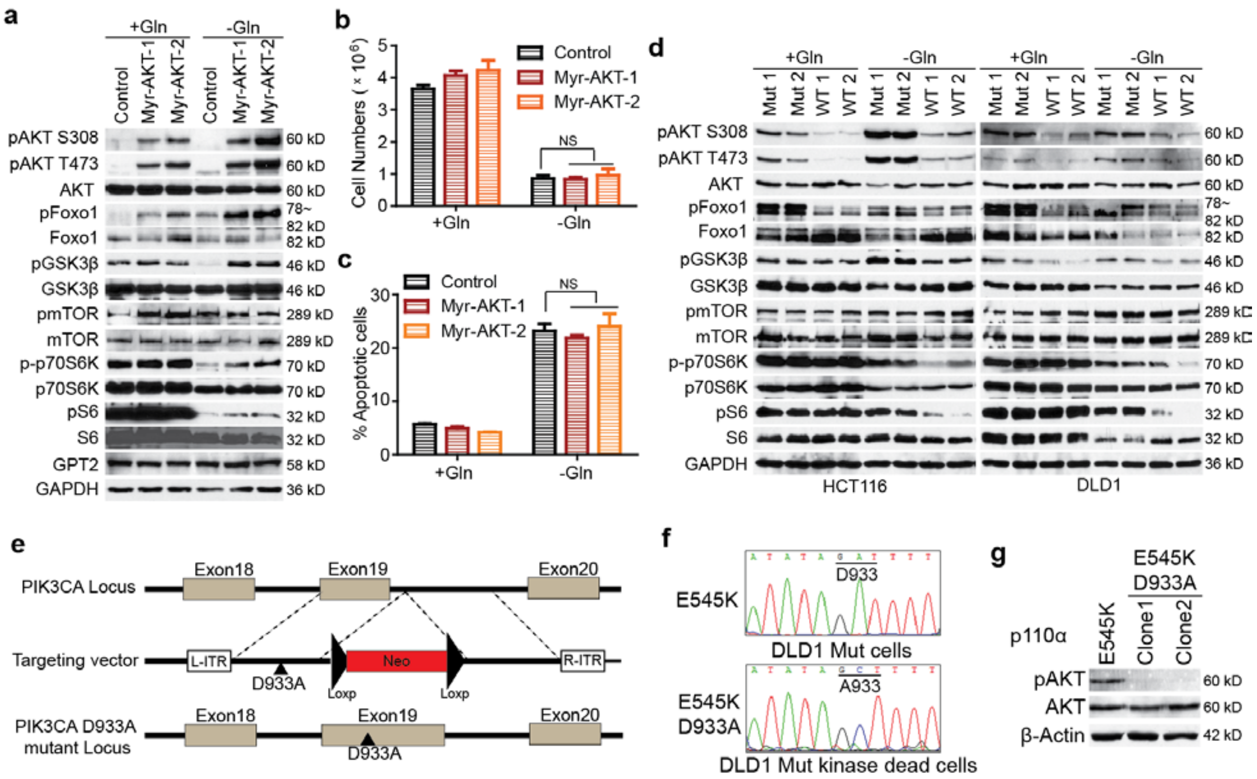

Supplementary Figure 5. Mutant p110α regulates ATF4 protein levels in an AKT-independent manner.

(a) to (**c**) Overexpression of constitutively active AKT does not sensitize HCT116 PIK3CA WT cells to glutamine deprivation. Myristoylated-Akt construct was transfected into HCT116 PIK3CA WT cells and two stable clones were selected. Cells were growth with or without glutamine for 72 hours. Cell lysates were blotted with the indicated antibodies (a). Viable cells were counted (b) and apoptotic cells were quantified (c).

(d) Phosphorylation levels of S6 are decreased in PIK3CA WT clones after glutamine deprivation. Clones of the indicated genotypes were grown with or without glutamine for 48 hours. Cell lysates were blotted with the indicated antibodies.

(e) Schematic of knockin of D933A lipid kinase killing mutation into a PIK3CA E545K mutant clone using adeno-associated virus-mediated gene targeting.

(f) Genomic sequences of parental and a E545K/D933A knockin clone.

(g) E545K D933A double mutant reduces AKT phosphorylation.

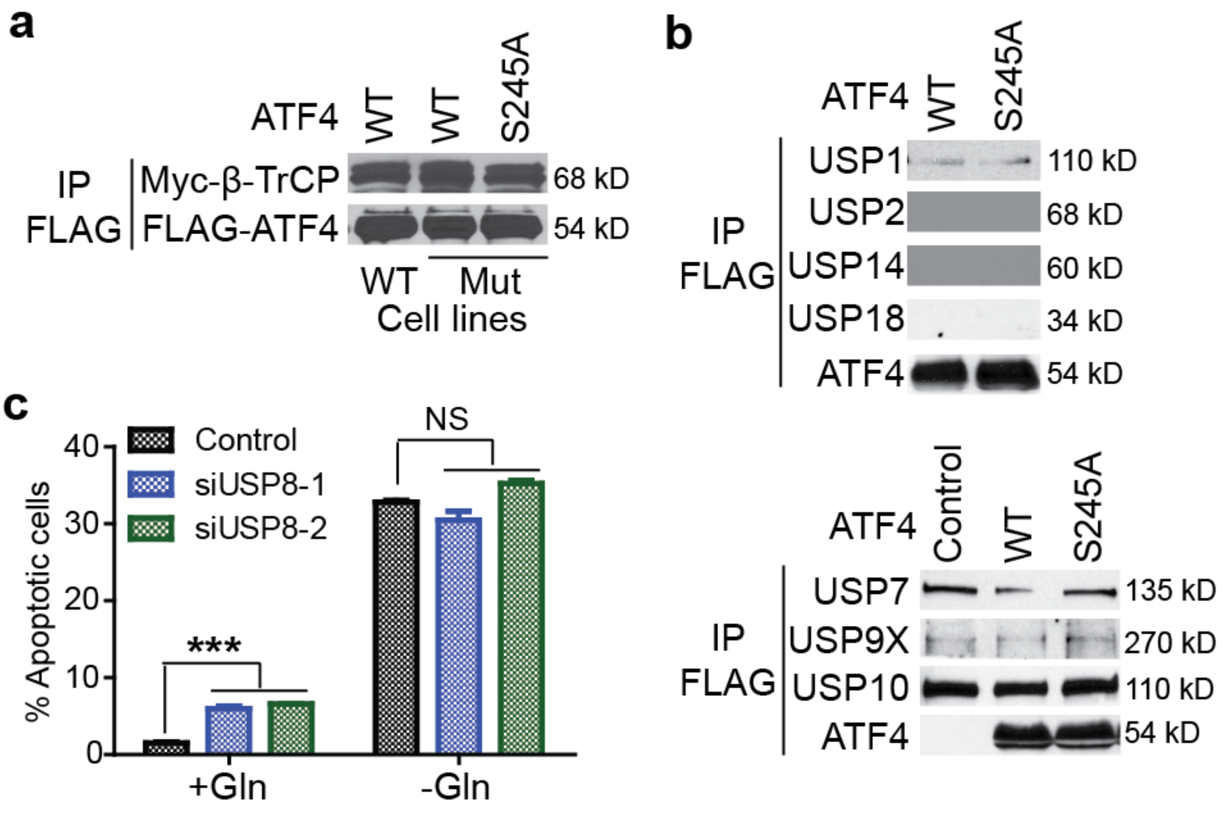

Supplementary Figure 6. ATF4 and USP8 association is specific.

(a) WT and S245A mutant ATF4 bind to similar amounts of ubiquitin E3 ligase β-TrCP1. Flag-tagged WT or S245A ATF4 and Myc-tagged β-TrCP1 expression vectors were co-transfected into the indicated HCT116 PIK3CA clones. Cell lysates were immunoprecipitated with anti-FLAG antibodies and immunocomplexes were blotted with an anti-Myc antibody.

(b) ATF4 does not associate with USP1, USP2, USP7, USP9X, USP10, USP14 or USP18. Flag-tagged ATF4 WT, S245A mutant or empty vector were transfected into the HCT116 PIK3CA mutant clone. Cell lysates were immunoprecipitated with anti-FLAG antibodies and blotted with the indicated anti-USP antibodies.

(c) Knockdown of USP8 in HCT116 PIK3CA mutant cells increases apoptosis in the presence of glutamine. HCT116 PIK3CA mutant cells were transfected with control siRNA or two independent siRNAs of USP8. Forty-eight hours post-transfection, cells were grown with or without glutamine for 72 hours. Apoptotic cells were quantified.

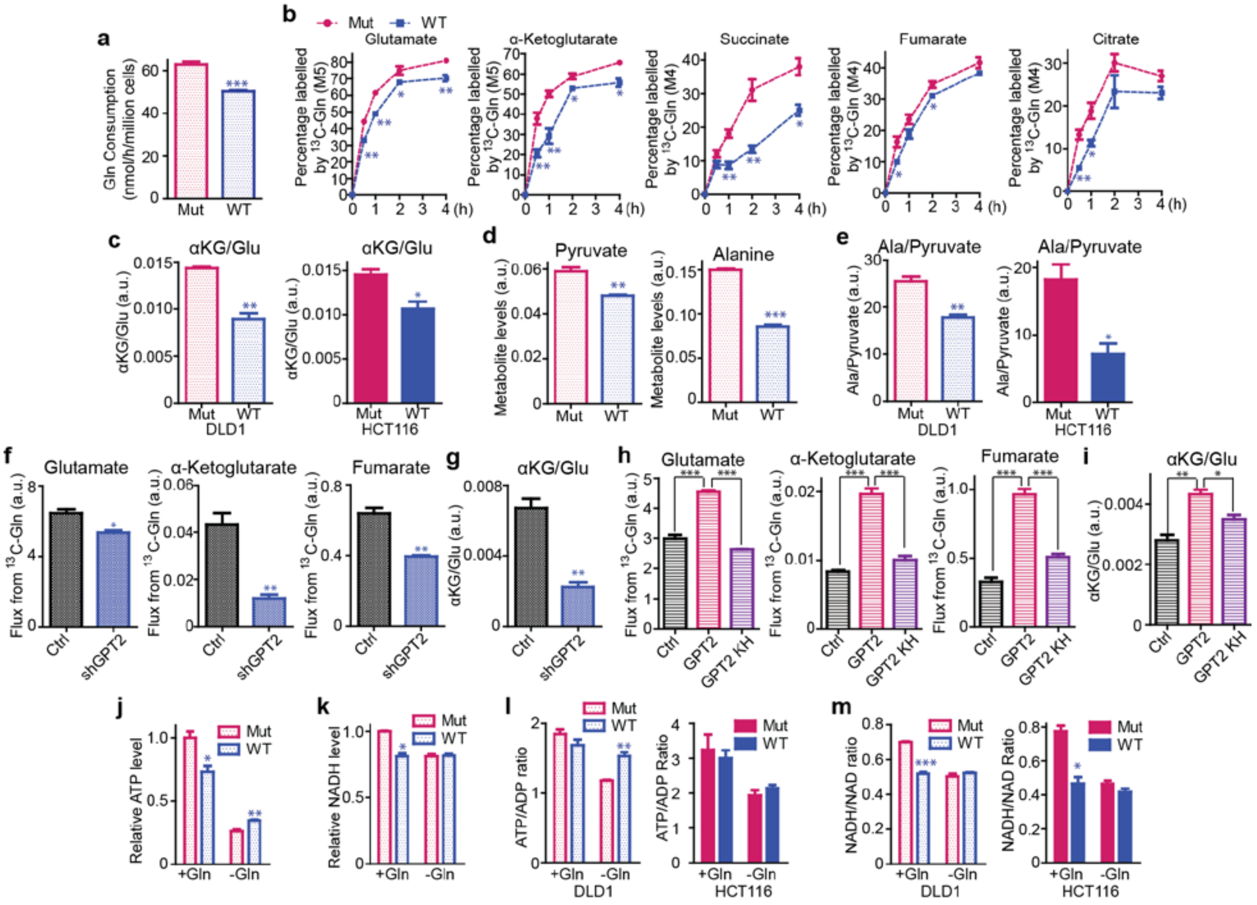

Supplementary Figure 7. PIK3CA mutant utilize more glutamine and generate more ATP.

(a) Glutamine consumption is greater in DLD1 PIK3CA mutant than WT clones. Cells were cultured with 2 mM initial concentration of [$^{13}C_5$]glutamine for 24 hours. Concentration of leftover [$^{13}C_5$]glutamine were measured by GC-MS to calculate glutamine consumption.

(**b**) The flux of glutamine-derived TCA cycle intermediates are higher in DLD1 PIK3CA mutant cells than in the isogenic WT cells. Cells were cultured in the presence of 2 mM [$^{13}C_5$]glutamine and direct glutamine-derived metabolites were measured by GC-MS at the indicated time points. Percentages of each labeled metabolite in total pool over the time course were plotted.

(**c**) Ratios of glutamine-derived α-KG/glutamate (Glu) are higher in PIK3CA mutant clones than the WT counterparts.

(**d**) Intracellular pyruvate and alanine levels are higher in DLD1 PIK3CA mutant cells than in the WT cells. Total amounts of the indicated metabolite (unlabeled) were measured by GC-MS and then normalized to internal standards and cell numbers.

(**e**) Ratios of intracellular alanine/pyruvate are higher in PIK3CA mutant clones than the WT counterparts.

(**f**) & (**g**) Knockdown of GPT2 reduces the ratio of glutamine-derived α-KG/glutamate in HCT116 PIK3CA mutant cells. Control and GPT2 knockdown cells were cultured in the presence of 2 mM [$^{13}C_5$]glutamine and direct glutamine-derived metabolites are measured by GC-MS at 2 hours (g). Ratios of glutamine-derived α-KG/glutamate are calculated (h).

(**h**) & (**i**) Overexpression of WT, but not enzymatically inactive mutant GPT2, in HCT116 PIK3CA WT cells increases the ratio of glutamine-derived α-KG/glutamate.

(**j**) & **(k**) Relative levels of ATP and NADH in the DLD1 PIK3CA WT and mutant clones with or without glutamine. ATP (k); NADH (l).

(l) The indicated clones were cultured with or without glutamine. The ratios of ATP/ADP were measure in each conditions.

(m) The ratios of NADH/NAD were measured in the indicated conditions.

Data are presented as mean $\pm$ SEM of three independent cultures. * $p < 0.05$; ** $p < 0.01$; *** $p < 0.001$; t test.

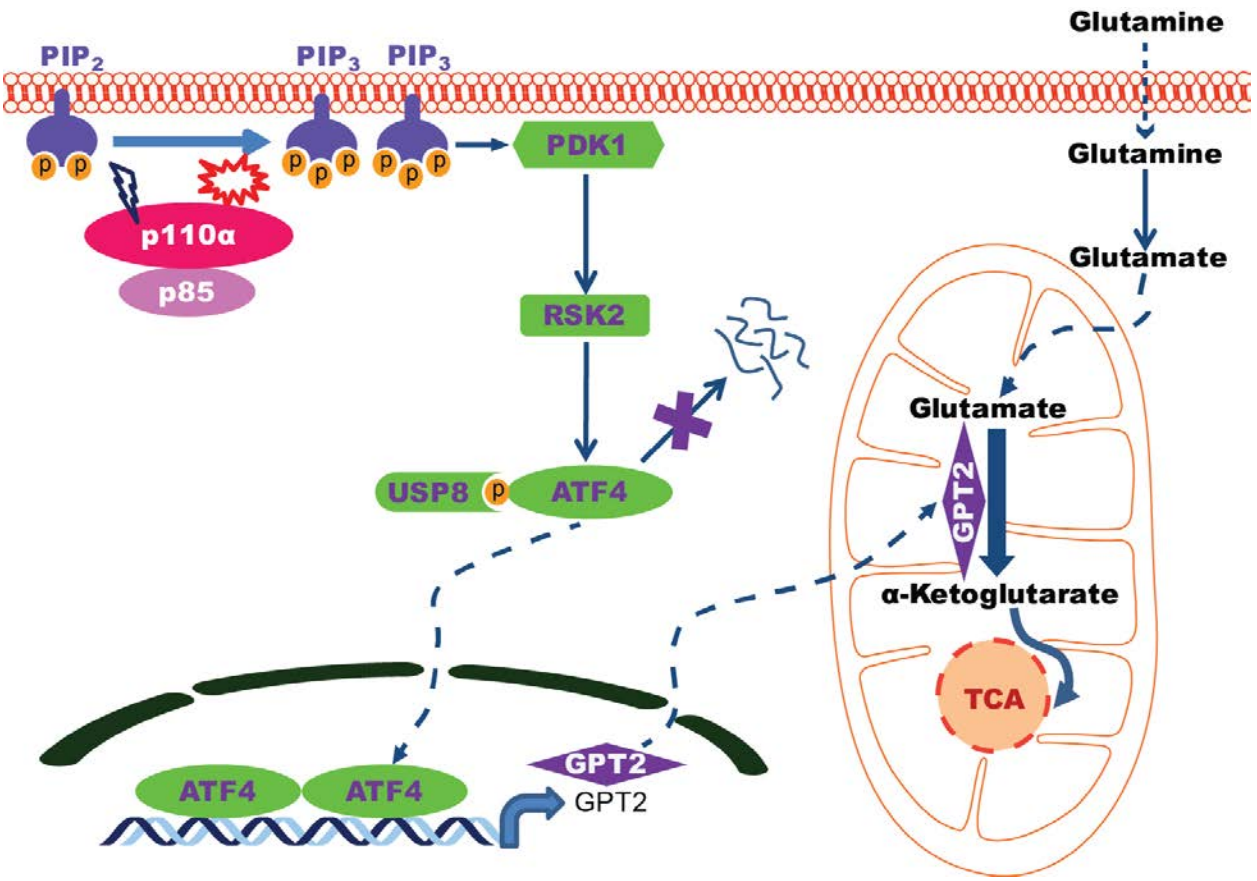

Supplementary Figure 8. A model for how oncogenic PIK3CA mutations render colorectal cancer cells dependent on glutamine.

Supplementary Figure 9. Original images of Western blot and Agarose gels.

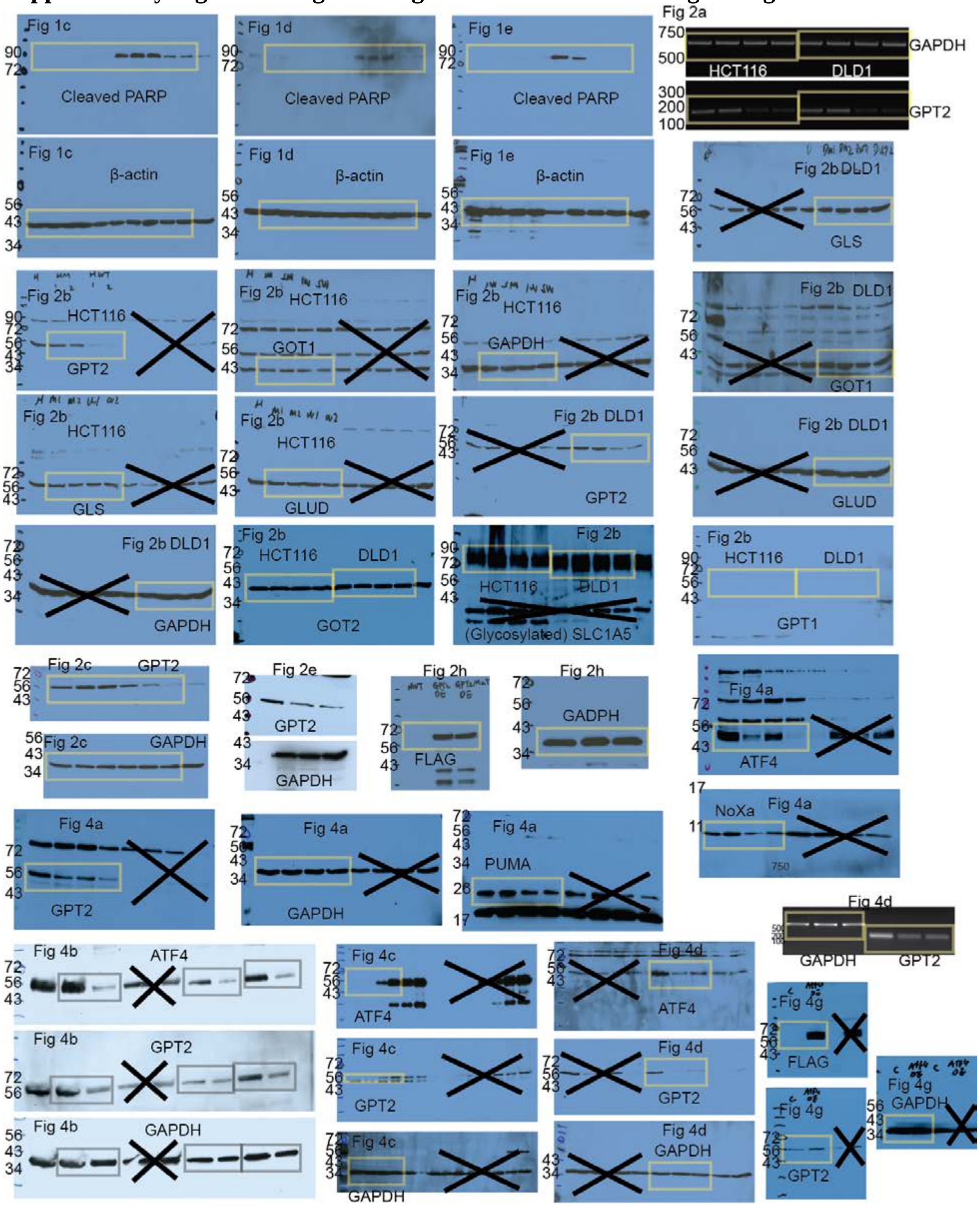

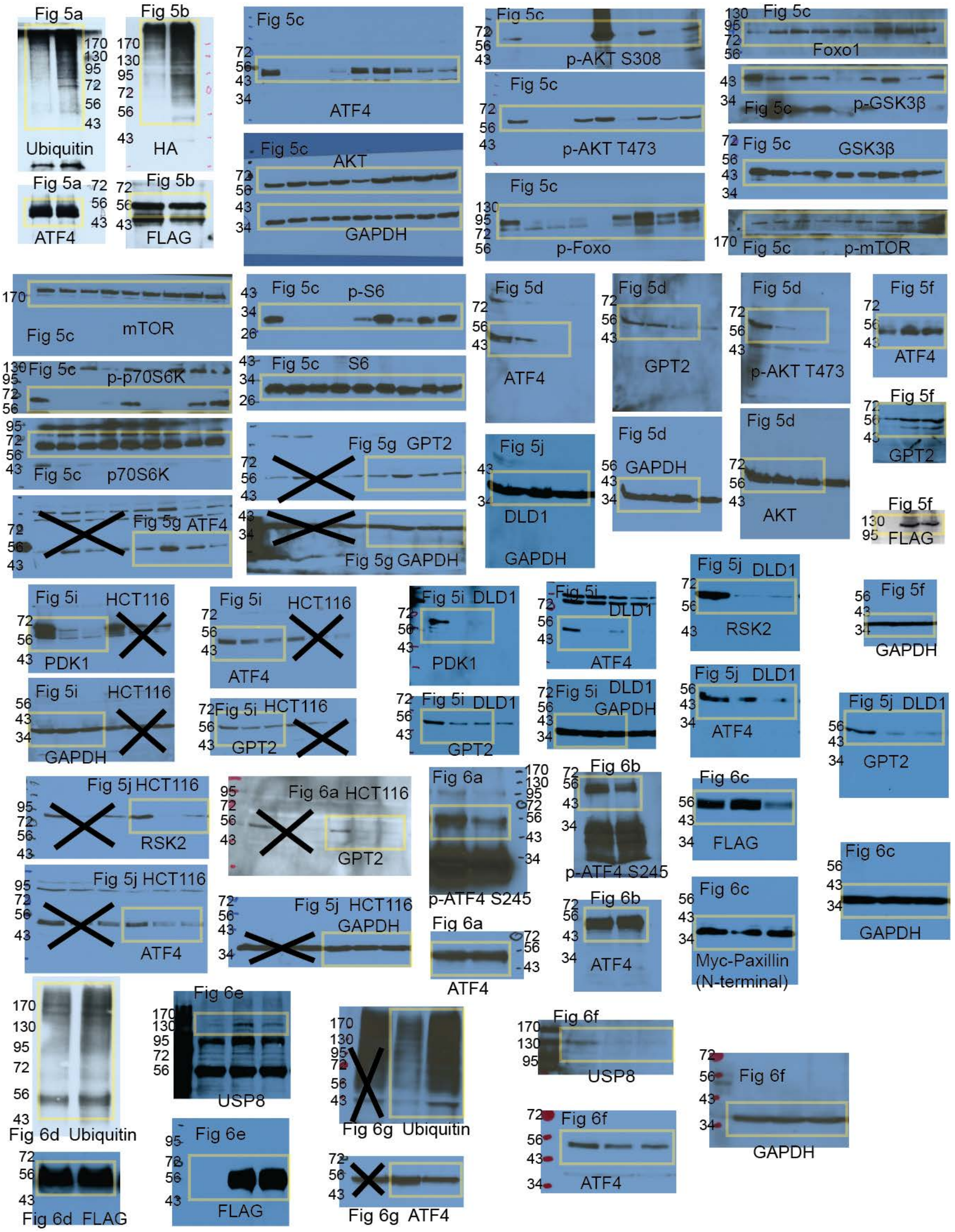
Fig 5a
Ubiquitin
Fig 5a
ATF4
Fig 5b
HA
Fig 5b
FLAG
Fig 5c
ATF4
Fig 5c
AKT
GAPDH
Fig 5c
p-AKT S308
Fig 5c
p-AKT T473
Fig 5c
p-Foxo
Fig 5c
Foxo1
Fig 5c
p-GSK3β
Fig 5c
GSK3β
Fig 5c
p-mTOR
Fig 5c
mTOR
Fig 5c
p-S6
Fig 5c
S6
Fig 5c
p-p70S6K
Fig 5c
p70S6K
Fig 5g
GPT2
Fig 5g
ATF4
Fig 5g
GAPDH
Fig 5d
ATF4
Fig 5d
GPT2
Fig 5d
p-AKT T473
Fig 5d
GAPDH
Fig 5d
AKT
Fig 5j
DLD1
GAPDH
Fig 5f
ATF4
Fig 5f
GPT2
Fig 5f
FLAG
Fig 5i HCT116
PDK1
Fig 5i HCT116
ATF4
Fig 5i DLD1
PDK1
Fig 5i DLD1
ATF4
Fig 5j DLD1
RSK2
Fig 5f
GAPDH
Fig 5i HCT116
GAPDH
Fig 5i HCT116
GPT2
Fig 5i DLD1
GPT2
Fig 5i DLD1
GAPDH
Fig 5j DLD1
ATF4
Fig 5j DLD1
GPT2
Fig 5j HCT116
RSK2
Fig 6a HCT116
GPT2
Fig 6a
p-ATF4 S245
Fig 6b
p-ATF4 S245
Fig 6c
FLAG
Fig 6c
GAPDH
Fig 5j HCT116
ATF4
Fig 5j HCT116
GAPDH
Fig 6a
ATF4
Fig 6b
ATF4
Fig 6c
Myc-Paxillin
(N-terminal)
Fig 6d Ubiquitin
Fig 6e
USP8
Fig 6g Ubiquitin
Fig 6f
USP8
Fig 6f
GAPDH
Fig 6f
ATF4
Fig 6d FLAG
Fig 6e
FLAG
Fig 6g ATF4

Supplementary Table 1. Antibodies used in this study.

Antibodies	Application	Company	Catalog number	Dilution
GPT2	IB	Proteintech Group	16757-1-AP	1:600
GLS1	IB	Proteintech Group	20170-1-AP	1:1000
GPT1	IB	Proteintech Group	16897-1-AP	1:600
GOT1	IB	Proteintech Group	14886-1-AP	1:1000
GOT2	IB	Proteintech Group	14800-1-AP	1:1000
GLUD	IB	Proteintech Group	14299-1-AP	1:1000
SLC1A5	IB	Abcam	Ab84903	1:1000
PCK2	IB	Abcam	Ab137580	1:1000
ATF4	IB, IP	Santa Cruz	sc-200	IB 1:500 IP 1:100
HA	IB	Santa Cruz	sc-805	1:1000
β-actin	IB	Sigma	A5441	1:5000
GAPDH	IB	Santa Cruz	sc-25778	1:2000
c-myc	IB	Santa Cruz	sc-40	1:1000
PUMA	IB	Cell Signaling	4976S	1:1000
p-AKT S308	IB	Cell Signaling	2965	1:1000
p-AKT T473	IB	Cell Signaling	4060	1:1000
AKT	IB	Cell Signaling	9272	1:1000
p-GSK3β	IB	Cell Signaling	9336	1:1000
GSK3β	IB	Cell Signaling	9315	1:1000
p-mTOR	IB	Cell Signaling	5536	1:1000
mTOR	IB	Cell Signaling	2983	1:1000
p-p70S6K	IB	Cell Signaling	9208	1:1000
p70S6K	IB	Cell Signaling	2708	1:1000
p-S6	IB	Cell Signaling	5364	1:1000
S6	IB	Cell Signaling	2217	1:1000
p-Foxo	IB	Cell Signaling	9464S	1:1000
RSK2	IB	Cell Signaling	5528S	1:1000
p-eIF2α	IB	Cell Signaling	3398S	1:1000
eIF2α	IB	Cell Signaling	9722	1:1000
Ubiquitin	IB	Cell Signaling	3936	1:500
USP8	IB	Cell Signaling	8728	1:600
USP7	IB	Cell Signaling	4833	1:600
USP1	IB	Cell Signaling	8033	1:600
USP2	IB	Cell Signaling	8036	1:600
USP9X	IB	Cell Signaling	5751	1:600
USP10	IB	Cell Signaling	8501	1:600
USP14	IB	Cell Signaling	8159	1:600
USP18	IB	Cell Signaling	4813	1:600
Cleaved PARP	IB	Cell Signaling	9544	1:1000
Foxo1	IB	Millipore	05-1075	1:500
pATF4 S245	IB	Abcam	ab28830	1:500
PDK1	IB	Abcam	ab52893	1:600
FLAG	IB	Sigma	F1804	1:500

Supplementary Table 2. Primers used in this study

**Targeting primers**		
	Left Arm forward	GGGAAAG/ideoxyU/GATGAGTCTGTCGGTGTTTGTG
	D933A reverse	AAAGCTATATGAAACAGCTTTCAAA
	D933A forward	TTTGAAAGCTGTTTCATATAGCTTTTGGACACTTTTTGGATC
	Left Arm reverse	GGAGACA/ideoxyU/TTTTGTGTTTTTAATTGCTCGAGC
p110α D933A	Right Arm forward	GGTCCCA/ideoxyU/CTGGCTGCTCTATTAGAAACAATC
Knock-In vector	Right Arm reverse	GGCATAG/ideoxyU/GATGTTGACATGGATGTGGTGA
	Screening forward	CTGCAGTTCAACAGCCACAC
	Screening reverse	CAGGGAAATGCAAATTAAAACC
	Cre forward	GTAAAGGAGCCCAAGAATGC
	Cre reverse	GCCAACATTTATTATTTTGAAATTG
**Subcloning primers**		
GPT2	Forward	CCCAAGCTTATGCAGCGGGCGGCGGCGC
pCMV-3Tag1A	Reverse	ACGCGTCGACTCACGCGTACTTCTCCAGGAAG
Flag-GPT2	Forward	CGGGGTACCGCCACCATGGATTACAAGGATGACGACG
pCDNA3.1zeo	Reverse	TGCTCTAGATCACGCGTACTTCTCCAGGAAG
FLAG-ATF4	Forward	CGCGGATCCATGACCGAAATGAGCTTCCTGAG
pCMV-3Tag1A	Reverse	CCGCTCGAGCTAGGGGACCCTTTTCTTCC
Myc-β-TrCP	Forward	GGTCCCA/ideoxyU/TGGACCCGGCCGAGGCGGTG
pCMV-Tag2	Reverse	GGCATAG/ideoxyU/TCTGGAGATGTAGGTGTATG
GPT2 promoter	Forward	CGGGGTACCCTGGGGAAGACTTTTACCTA
pGL3	Reverse	GGAAGATCTCCACAGCCGCATCCCCGCGC
GPT2 sgRNA	Forward	CACCGCACAAGCCTGCAGGATCCGC
GPT2 sgRNA	Reverse	AAACGCGGATCCTGCAGGCTTGTGC
**Mutagenesis primers**		
FLAG-GPT2 K341H	Forward	CCTTCCACTCCACCTCCCACGGCTACATGGGCGAGTG
	Reverse	CACTCGCCCATGTAGCCGTGGGAGGTGGAGTGGAAGG

FLAG-ATF4 S219A	Forward	CTTCAGATAATGATGCTGGCATCTGTATGAGC
	Reverse	GCTCATACAGATGCCAGCATCATTATCTGAAG
FLAG-ATF4 S245A	Forward	CAGGGGCTCTCCAAATAGGGCGCTCCCATCTCCAGGTGTTC
	Reverse	GAACACCTGGAGATGGGAGCGCCCTATTTGGAGAGCCCCTG
GPT2 promoter	Forward	CGGAAGTGATGGAGGTCGTTGCGCTAATGGAGTGGTCGGGAAAAC
Mut1	Reverse	GTTTTCCCGACCACTCCATTAGCGCAACGACCTCCATCACTTCCG
GPT2 promoter	Forward	GCACCGTGTGGCCTTGGAGTTGCGCTACTCGGGGCGATGACTGCAC
Mut2	Reverse	GTGCAGTCATCGCCCCGAGTAGCGCAACTCCAAGGCCACACGGTGC
**RT-PCR primers**		
SLC1A5	Forward	CATCATCCTCGAAGCAGTCA
	Reverse	CTCCGTACGGTCCACGTAAT
GLS1	Forward	TGCATTCCTGTGGCATGTAT
	Reverse	TTGCCCATCTTATCCAGAGG
GPT2	Forward	CTTTCTCCTGGCTGATGAGG
	Reverse	TAACCACACTCGCCCATGTA
GOT1	Forward	ACCTGGGAGAATCACAATGC
	Reverse	GCGGCTGTGCCCGCCGGTGC
GOT2	Forward	CAATGGCTGCAAGAAGTGAA
	Reverse	GGCTTTAGCCCTGTGAAACA
GLUD1	Forward	CACACGCCTGTGTTACTGGT
	Reverse	CTCCAAACCCTGGTGTCATT
GAPDH	Forward	GGAAATCCCATCACCATCT
	Reverse	TGTCGCTGTTGAAGTCAGA
ATF4	Forward	CCAACAACAGCAAGGAGGAT
	Reverse	AGTGTCATCCAACGTGGTCA

Supplementary Table 3. Tumor samples of Quantitative Real-Time PCR

Tumors	PIK3CA mutation	Tumors	Mutation in PI3K pathway
435X	PIK3CA E545K	560X	WT
507X	PIK3CA E545K	569X	WT
533X	PIK3CA H1047R	492X	WT
511X	PIK3CA Q546K	452X	WT
587X	PIK3CA H1047R	493X	WT
480X	PIK3CA R38C	566X	WT
579X	PIK3CA H1047R	586X	WT
823X	PIK3CA H1047R	559X	WT
X841	PIK3CA H1047R	464X	WT
X850	PIK3CA E542K	502X	WT
